# Acetaminophen as a Renoprotective Adjunctive Treatment in Patients With Severe and Moderately Severe Falciparum Malaria: A Randomized, Controlled, Open-Label Trial

**DOI:** 10.1093/cid/ciy213

**Published:** 2018-03-12

**Authors:** Katherine Plewes, Hugh W F Kingston, Aniruddha Ghose, Thanaporn Wattanakul, Md Mahtab Uddin Hassan, Md Shafiul Haider, Prodip K Dutta, Md Akhterul Islam, Shamsul Alam, Selim Md Jahangir, A S M Zahed, Md Abdus Sattar, M A Hassan Chowdhury, M Trent Herdman, Stije J Leopold, Haruhiko Ishioka, Kim A Piera, Prakaykaew Charunwatthana, Kamolrat Silamut, Tsin W Yeo, Sue J Lee, Mavuto Mukaka, Richard J Maude, Gareth D H Turner, Md Abul Faiz, Joel Tarning, John A Oates, Nicholas M Anstey, Nicholas J White, Nicholas P J Day, Md Amir Hossain, L Jackson Roberts II, Arjen M Dondorp

**Affiliations:** 1 Mahidol Oxford Tropical Medicine Research Unit, Mahidol University, Bangkok, Thailand; 2 Centre for Tropical Medicine and Global Health, Nuffield Department of Clinical Medicine, University of Oxford, United Kingdom; 3 Department of Medicine, University of British Columbia, Vancouver, Canada; 4 Menzies School of Health Research, Charles Darwin University, Northern Territory, Australia; 5 Department of Medicine, Chittagong Medical College Hospital; 6 Department of Nephrology, Chittagong Medical College Hospital; 7 Ramu Upazilla Health Complex, Cox’s Bazaar; 8 Department of Anesthesiology, Chittagong Medical College Hospital; 9 Department of Pharmacology, Chittagong Medical College Hospital; 10 Department of Clinical Tropical Medicine, Mahidol University, Bangkok, Thailand; 11 Lee Kong Chian School of Medicine, Nanyang Technological University, Singapore; 12 Harvard TH Chan School of Public Health, Harvard University, Boston, Massachusetts; 13 Malaria Research Group, and Dev Care Foundation, Dhaka, Bangladesh; 14 Department of Internal Medicine, Vanderbilt University School of Medicine, Nashville,Tennessee

**Keywords:** falciparum malaria, acute kidney injury, cell-free hemoglobin, oxidative stress, acetaminophen

## Abstract

**Background:**

Acute kidney injury independently predicts mortality in falciparum malaria. It is unknown whether acetaminophen’s capacity to inhibit plasma hemoglobin-mediated oxidation is renoprotective in severe malaria.

**Methods:**

This phase 2, open-label, randomized controlled trial conducted at two hospitals in Bangladesh assessed effects on renal function, safety, pharmacokinetic (PK) properties and pharmacodynamic (PD) effects of acetaminophen. Febrile patients (>12 years) with severe falciparum malaria were randomly assigned to receive acetaminophen (1 g 6–hourly for 72 hours) or no acetaminophen, in addition to intravenous artesunate. Primary outcome was the proportional change in creatinine after 72 hours stratified by median plasma hemoglobin.

**Results:**

Between 2012 and 2014, 62 patients were randomly assigned to receive acetaminophen (n = 31) or no acetaminophen (n = 31). Median (interquartile range) reduction in creatinine after 72 hours was 23% (37% to 18%) in patients assigned to acetaminophen, versus 14% (29% to 0%) in patients assigned to no acetaminophen (*P* = .043). This difference in reduction was 37% (48% to 22%) versus 14% (30% to −71%) in patients with hemoglobin ≥45000 ng/mL (*P* = .010). The proportion with progressing kidney injury was higher among controls (subdistribution hazard ratio, 3.0; 95% confidence interval, 1.1 to 8.5; *P* = .034). PK–PD analyses showed that higher exposure to acetaminophen increased the probability of creatinine improvement. No patient fulfilled Hy’s law for hepatotoxicity.

**Conclusions:**

In this proof-of-principle study, acetaminophen showed renoprotection without evidence of safety concerns in patients with severe falciparum malaria, particularly in those with prominent intravascular hemolysis.

**Clinical Trials Registration:**

NCT01641289.

## Teaser

This randomized, controlled trial shows that acetaminophen reduces kidney dysfunction and risk of developing acute kidney injury, particularly in severe malaria patients who present with high plasma hemoglobin, supporting the hypothesis that acetaminophen inhibits cell-free hemoglobin-mediated renal tubular oxidative damage.

Kidney dysfunction complicating severe falciparum malaria is common and independently predicts mortality in all age groups [1, 2]. Mortality in patients with severe acute kidney injury (AKI) is approximately 75% without and 26% with renal replacement therapy [3], but this therapy is frequently unavailable in malaria-endemic areas.

Intravascular hemolysis that results in high levels of plasma cell-free hemoglobin (CFH) contributes to AKI in several conditions, including post-cardiopulmonary bypass [4], paroxysmal nocturnal hemoglobinuria [5], and massive transfusion [6]. After haptoglobin depletion, CFH causes oxidative renal tubular damage through redox cycling between heme-ferric and ferryl states, which results in lipid peroxidation generating F_2_-isoprostanes (F_2_-IsoPs) and isofurans (IsoFs) [7, 8]. F_2_-IsoPs are potent renal vasoconstrictors that act via thromboxane A_2_ receptors [8]. Intravascular hemolysis is an intrinsic component of severe falciparum malaria pathophysiology [9]. Recently it was shown that increased levels of CFH, F_2_-IsoPs, and IsoFs are strongly associated with kidney dysfunction and hemodialysis requirement in adults with severe malaria [10]. Acetaminophen inhibits hemoprotein-mediated lipid peroxidation by reducing heme-ferryl radicals [11]. In an experimental rhabdomyolysis model, acetaminophen decreased oxidative stress markers and attenuated AKI [11].

We conducted a randomized, controlled trial of acetaminophen vs no acetaminophen in patients with severe and moderately severe malaria to assess acetaminophen as a renoprotective adjunctive therapy. We hypothesized that adjunctive therapy with acetaminophen would improve kidney function, particularly in patients with prominent intravascular hemolysis.

## METHODS

### Trial Design

The study was a multicenter, randomized, open-label, controlled clinical trial among patients admitted at 2 hospitals in southeastern Bangladesh, Thailand: Ramu Upazilla Health Complex (primary subdistrict hospital) and Chittagong Medical College Hospital (CMCH; tertiary referral hospital).

### Participants

Eligible patients were aged >12 years with microscopy-confirmed *Plasmodium falciparum* severe or moderately severe malaria (Supplementary Table 1) and fever (>38°C on admission or fever during preceding 24 hours). Written informed consent was obtained from the patient or a legally acceptable representative if the patient was unconscious. Severe malaria was defined as presence of asexual parasitemia plus 1 or more severity criteria [12]. Moderate disease was defined as the need for parenteral therapy without a severity criterion. Exclusion criteria were pregnancy, history of chronic liver disease or alcohol abuse, and contraindications for acetaminophen or nasogastric (NG) tube insertion.

### Randomization, Masking, and Intervention

Randomization to acetaminophen or control was stratified by severity of malaria using a computerized binary sequence number generator in a 1:1 ratio (blocks of 20), generated by a statistician unrelated to the study. Opaque, sealed envelopes that contained treatment allocations were opened by a research physician after enrollment. Laboratory staff performed quantification of the primary endpoint. All biochemical tests were masked to the treatment allocation; research physicians and study participants were not. Study codes on samples were nonidentifiable for drug allocation. The study data manager was responsible for data entry, cleaning, and extraction.

Acetaminophen was given as observed therapy at a 6-hourly dose of 1 g (patient weight ≥50 kg) or 12.5–15.0 mg/kg/dose (<50 kg) for 72 hours, as tablets (500 mg, Bristol Laboratories Ltd., UK) to conscious patients or as syrup (250 mg/5 mL, Rosemont Pharmaceuticals Ltd., UK) via NG tube to comatose patients. This is the standard recommended dosage [12] to achieve therapeutic levels for antipyresis [13] and renoprotection [11, 14]. If patients vomited within 1 hour, the dose was repeated. In the control arm, patients with fever >40°C were given oral ibuprofen 400 mg (or diclofenac suppository 50 mg) or 500 mg acetaminophen in case of renal impairment, dehydration, or dengue infection. Antimalarial treatment was with intravenous artesunate (Guilin Pharmaceuticals, China), followed by artemether/lumefantrine (Coartem, Novartis, Switzerland) once oral medication was tolerated. Supportive management was according to World Health Organization guidelines [12]. Patients without shock, anuria, or severe dehydration received intravenous normal saline at a rate of 250 mL/h for 6 hours followed by 100 mL/hour until oral fluids were tolerated. In case of severe dehydration with oliguria, the rate was increased to 1000 mL/hour for 2 hours followed by 500 mL/hour for 2 hours if oliguria persisted without signs of fluid overload. Patients in Ramu who required hemodialysis were transferred to CMCH. Hemodialysis was initiated by attending nephrologists not involved in data collection or analysis.

### Outcomes

The primary outcome was the relative change in serum creatinine at 72 hours from enrollment stratified by enrollment CFH concentration. Secondary outcomes included proportion of patients developing AKI according to Kidney Disease: Improving Global Outcomes (KDIGO) criteria (creatinine increase ≥26.5 µmol/L within 48 hours) [15]; fever clearance time (time until temperature <37.5°C [FCT–A] and time until temperature was <37.5°C for 24 hours [FCT–B]); parasite clearance (time to first of 2 consecutive negative slides and parasite half–life) [16]; and safety (in particular, hepatological parameters at 72 hours). Population pharmacokinetic (PK) properties and pharmacodynamic (PD) effects on parasitemia, temperature, and creatinine were characterized using nonlinear mixed-effects modeling, outlined in the Supplementary Material. AKI was staged at enrollment by KDIGO criteria [15], using an estimated baseline creatinine obtained from the Modification of Diet in Renal Disease formula if aged ≥19 years (glomerular filtration rate [GFR], 75 mL/min/1.73 m^2^) and the bedside Schwartz formula if <19 years (GFR, 100 mL/min/1.73 m^2^) [15].

### Laboratory Methods

Screening venous blood samples were analyzed for parasitemia and biochemistry (point-of-care iSTAT analyzer [CG4+, Chem8+], Abbott Laboratories, USA). After enrollment, vital signs, urine output, development of complications, and parasitemia were assessed every 6 hours until discharge or death. For the first 72 hours, serum creatinine was assessed every 12 hours (Olympus AU400 chemistry analyzer, performed in Bangkok), CFH in twice-centrifuged citrated plasma was assessed every 24 hours (enzyme-linked immunosorbent assay; Bethyl Laboratories, performed in Darwin) [9], and F_2_-IsoPs and IsoFs in lithium heparin plasma were assessed every 24 hours (gas chromatography-mass spectrometry at Vanderbilt University) [7, 8]. Serum alanine aminotransferase (ALT) and aspartate aminotransferase (AST) were assessed on enrollment and at 72 hours. To determine the PK–PD of acetaminophen on creatinine, parasitemia, and fever, plasma EDTA samples for acetaminophen concentration were collected prior to each dose plus dense sampling in the treatment arm after both the first (0 hour) and last (72 hour) dose. Patients in the control arm had samples collected every 6 hour for 72 hours to assess unplanned acetaminophen intake. Detailed procedures are provided in the Supplementary Material.

### Statistical Analyses

Inclusion of 62 patients allowed demonstration of a difference in proportional change in serum creatinine at 72 hours of 10% with 5% significance and 90% power, assuming a mean (standard deviation) admission creatinine value of 150 μmol/L (90 μmol/L). The primary endpoint analysis was modified intention-to-treat (ITT) principle including all patients who were randomly assigned. Modified ITT was applied because it was challenging to impute and analyze missing 72-hour creatinine data since most of those with missing data died. Between-group differences were compared with Student *t* test or Wilcoxon-Mann-Whitney test for continuous and Fisher exact test for categorical variables. Primary endpoint comparison adjusted by enrollment CFH was analyzed by linear regression. Temporal data were analyzed using mixed effects models, with the maximum likelihood method of estimation. Stratification was by enrollment median CFH, F_2_-IsoP, and IsoF concentrations. Where interaction terms were significant, the lincom command was used to obtain overall treatment effects. Time-to-event outcome subdistribution hazard ratios (SHRs) were estimated by competing-risks regression to account for death as a competing event for the event of interest (AKI). All patients with a creatinine rise of ≥26.5 µmol/L after enrollment were included. Parasite and fever clearance times were assessed using Kaplan-Meier survival analyses. For missing creatinine values in the longitudinal series, totaling 35 of 434 (8%) values, multiple imputation using chained equations with 5 rounds was used (Supplementary Table 2) [17]. In the mixed effects analyses, it was assumed that in patients on hemodialysis, a creatinine rise of 132.6 µmol/L per day was averted, which is the rise proposed for anephric states [18].

The Oxford University Tropical Research Ethics Committee and Chittagong Medical College Ethics Committee approved the study protocol. Trial sites were monitored by the Clinical Trials Support Group from Mahidol Oxford Research Unit, Bangkok, Thailand, and study outcomes followed by a data and safety monitoring committee.

## RESULTS

### Study Population

**Figure 1.**
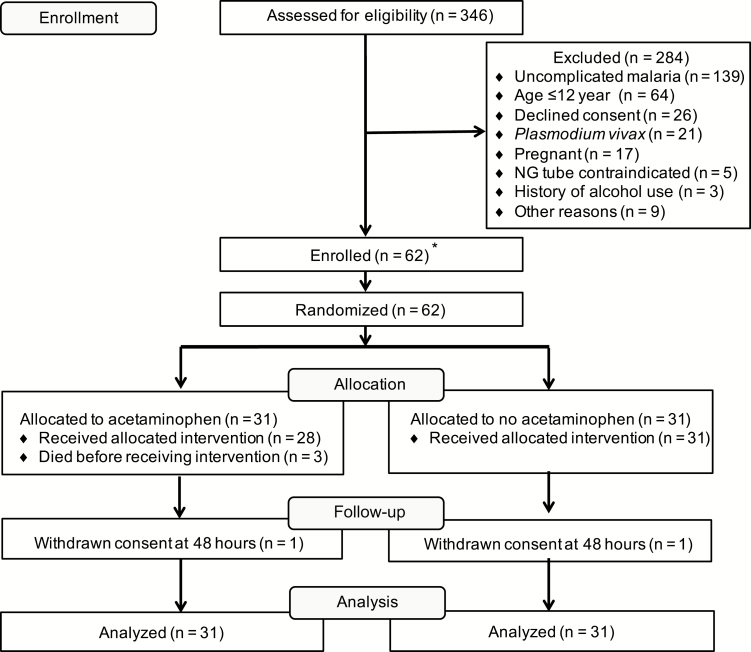
Flow chart for the “Acetaminophen as a Renoprotective Adjunctive Treatment in Patients With Severe and Moderately Severe Falciparum Malaria” study. *A total of 30 patients were recruited in Chittagong, Bangladesh, and 32 patients were recruited in Ramu, Bangladesh. Abbreviation: NG, nasogastric.

Recruitment was from 10 July 2012 until 11 September 2014. Of the 346 patients assessed, 62 were eligible for randomization to receive acetaminophen (n = 31) or no acetaminophen (n = 31). The trial profile is shown in Figure 1. Baseline characteristics were well matched between treatment arms (Tables 1 and 2). At enrollment, 42% (13/31) and 58% (18/31) had a CFH concentration above the median (45000 ng/mL) in the acetaminophen and control groups, respectively.

**Table 1.** Demographic and Baseline Clinical Characteristics by Treatment Arm

Characteristic	Acetaminophen (n = 31)	Control (n = 31)
Demographics		
Sex (male)	19 (61)	21 (68)
Age (years)	28 (20–43)	32 (25–40)
Fever before enrollment (days)	7 (4–10)	7 (5–8)
Altered level of consciousness before enrollment (days)	2.5 (1.0–3.0)	1.0 (1.0–2.5)
Red or black urine before enrollment	0 (0)	4 (13)
Acetaminophen before enrollment^a^	1 (3)	0 (0)
Comorbidities		
Hypertension	3 (10)	1 (3)
Coronary artery disease	3 (10)	1 (3)
Diabetes	1 (3)	0 (0)
Complications on enrollment		
Coma^b^	10 (32)	13 (42)
Jaundice	3 (10)	5 (16)
Severe anemia	2 (6)	2 (6)
Hyperlactatemia (lactate >4 mmol/L)	7 (23)	9 (29)
Hyperparasitemia (>10%)	1 (3)	4 (13)
Hemoglobinuria	2 (7)	6 (19)
Pulmonary edema	0 (0)	1 (3)
Severe prostration^c^	18 (58)	19 (61)
Unable to tolerate oral medications	22 (71)	24 (77)
Severity		
Severe malaria	24 (77)	25 (81)
Moderately severe malaria	7 (23)	6 (19)
Number of severity criteria	3 (1–5)	4 (1–6)
Kidney Disease: Improving Global Outcomes stage on enrollment		
0	17 (55)	17 (55)
1 (≥1.5 × baseline)	7 (23)	6 (19)
2 (≥2.0–2.9 × baseline)	3 (10)	3 (10)
3 (≥3 × baseline) OR (≥353.6 μmol/L)	4 (13)	5 (16)

Data are number (%) or median (interquartile range), unless otherwise indicated. There were no significant differences between treatment groups in any of the measured baseline characteristics.

^a^Acetaminophen before enrollment was based on an acetaminophen concentration >10 mg/L in enrollment pharmacokinetic samples.

^b^Depth of coma was assessed by Glascow coma score <11.

^c^Severe prostration defined as inability to walk or sit up without assistance.

**Table 2.** Baseline Clinical and Laboratory Investigations by Treatment Arm

Characteristic	Acetaminophen (n = 31)	Control (n = 31)
Clinical examination		
Temperature (°C)	39.0 (37.2–40.0)	38.9 (37.5–39.5)
Blood pressure (mmHg)		
Systolic	108 (100–116)	110 (95–119)
Diastolic	61 (50–70)	69 (59–75)
Glasgow coma score	15 (9–15)	12 (9–15)
Respiratory rate (breaths/minute)	30 (24–38)	30 (28–40)
Heart rate (beats/minute)	107 (96–118)	108 (92–130)
Laboratory investigations		
Parasite count per μL^a^	53426 (24596–116048)	17258 (5751–51785)
Plasma *Plasmodium falciparum* histidine rich protein 2 (mg/mL)	1356 (146–4486)	1308 (154–4961)
Sodium (mmol/L)	134 (130–138)	135 (130–138)
Potassium (mmol/L)	3.5 (3.3–4.0)	3.4 (3.0–3.9)
Chloride (mmol/L)	103 (99–107)	104 (100–107)
Glucose (mmol/L)	5.8 (4.7–8.1)	6.8 (5.5–9.0)
Blood urea nitrogen (mg/dL)	23 (13–36)	25 (16–43)
Creatinine (μmol/L)	106 (97–169)	115 (97–168)
Hemoglobin (g/dL)	10.1 (8.0–12.7)	11.6 (10.0–13.4)
Cell-free hemoglobin (ng/mL)	42800 (16800–94100)	52500 (23400–188800)
F_2_-isoprostanes (pg/mL)	22.0 (14.5–27.7)	23.6 (15.7–38.6)
Isofurans (pg/mL)	44.7 (25.0–64.2)	44.0 (26.1–84.0)
Total bilirubin (mg/dL)^a^	1.5 (1.0–2.2)	1.7 (1.2–2.4)
Indirect bilirubin (mg/dL)^a^	0.7 (0.5–0.9)	0.8 (0.6–1.2)
Lactate (mmol/L)	2.89 (1.94–3.70)	2.11 (1.62–4.84)
Bicarbonate (mmol/L)	19.2 (17.3–21.8)	18.5 (15.8–19.9)
Base excess (mmol/L)	-5 (-7–-3)	-7 (-10–-3)

Data are median (interquartile range), unless otherwise indicated by ^a^ geometric mean (95% confidence interval). There were no significant differences between treatment groups in any of the measured baseline characteristics.

### Outcomes

The median (interquartile range [IQR]) proportional reduction in serum creatinine at 72 hours compared to baseline was 23% (IQR, 37% to 18%) in patients receiving acetaminophen compared to 14% (IQR, 29% to 0%) in control patients (*P* = .043), which was more prominent when adjusted for enrollment CFH (*P* = .026; Supplementary Figure 2). In patients with high CFH at enrollment (≥45000 ng/mL), the median (IQR) reduction in creatinine at 72 hours was 37% (IQR, 48% to 22%) with acetaminophen vs 14% (IQR, 30 to −71%) in control patients (*P* = .010). The subgroup of patients with CFH <45000 ng/mL did not show a difference between treatment groups (*P* = .66). Creatinine concentrations over time are shown in Supplementary Tables 3 and 4. A total of 5 ibuprofen or diclofenac doses were administered to 4 patients (1–2 doses/patient) in the control arm, which was not associated with a rise in creatinine (Supplementary Figure 3).

**Figure 2.**
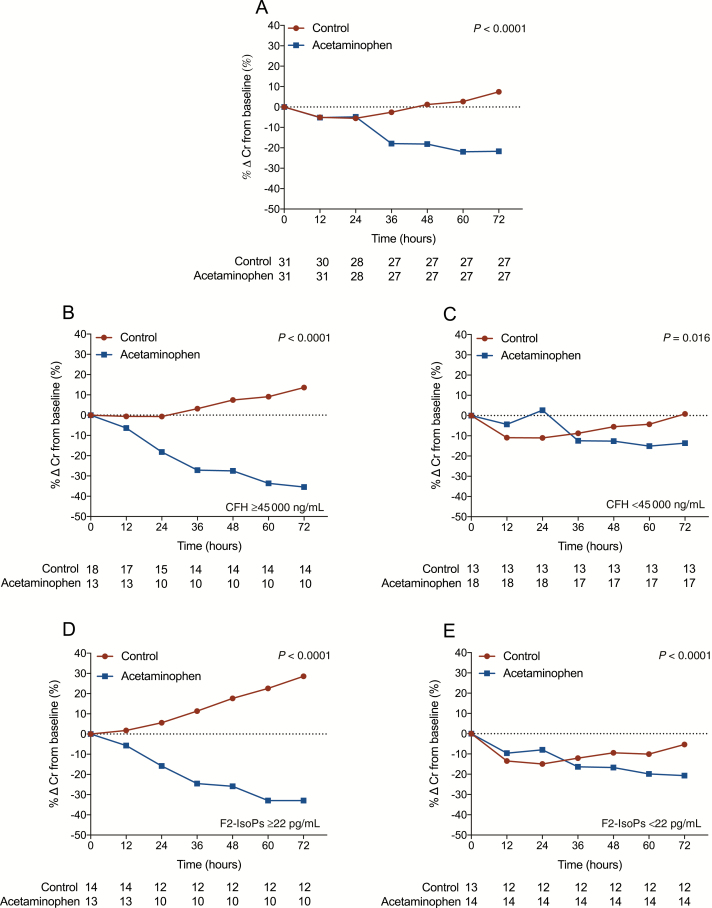
Effect of acetaminophen on creatinine stratified by intravascular hemolysis. Creatinine mean percent change from baseline at 12, 24, 36, 60, 48, and 72 hours of entire cohort *(A*) and patients stratified by level of intravascular hemolysis (*B* and *C*). Plasma cell-free hemoglobin (CFH) ≥45000 ng/mL *(B*); plasma CFH <45000 ng/mL *(C*); patients stratified by level of lipid peroxidation *(D* and *E*); plasma F_2_- isoprostanes (IsoPs) ≥22 pg/mL *(D*); plasma F_2_-IsoPs <22 pg/mL *(E*). A total of 35 of 434 (8%) creatinine sampling time points were missing and replaced by imputed values (for details see Supplementary Table 2). Frequencies in rows below figures represent number of patients (n) at each time point. *P* value represents overall treatment effect. Abbreviations: Cr, creatinine; CFH, cell-free hemoglobin; F_2_-IsoPs, F_2_-isoprostanes.

Mixed effects modeling using only the interaction term of treatment arm with time showed there was a significant interaction between treatment and time. In particular, creatinine improved over time in the group receiving acetaminophen but worsened over time in the control group (*P* < .001; Figure 2A; Supplementary Table 5). This difference was more pronounced in patients with high CFH at enrollment (*P* < .001; Figure 2B). Similarly, this beneficial effect of acetaminophen was more prominent in patients with increased F_2_-IsoPs and IsoFs on admission (*P* < .001; Figure 2D; Supplementary Figure 4*B*). In the patients with lower CFH and oxidative stress markers at enrollment, there was less difference in rates of creatinine change (Figure 2C and 2E; Supplementary Figure 4*C*). Mixed effects modeling using the interaction term of treatment group and CFH (as continuous variable), as well as the previously fitted treatment-time interaction term, showed there was a significant interaction between treatment and CFH. Specifically, the effect of acetaminophen on the reduction of creatinine depended on enrollment CFH (interaction *P* value = .016; Supplementary Figure 5 and Supplementary Table 5).

**Figure 3.**
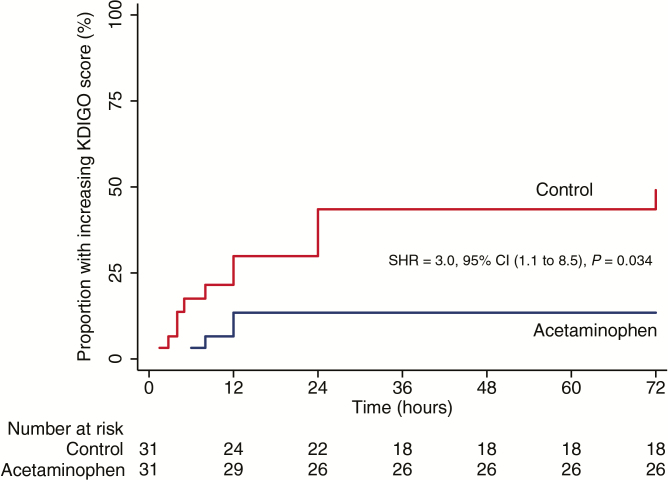
Kaplan–Meier plot comparing in-hospital acute kidney injury (AKI) development in patients with severe and moderately severe malaria treated with either acetaminophen or no acetaminophen (control). Patients were classified as developing AKI if they had a creatinine rise of ≥26.5 µmol/L after admission. A total of 17/62 (27%) patients had AKI during admission: 12/17 (38%) in the control group and 5/12 (16%) in the acetaminophen group. Competing risks regression adjusted by study site was used to assess subdistribution hazard ratio. Patients were censored at the time of creatinine rise meeting Kidney Disease: Improving Global Outcomes criteria and death censored as a competing risk preventing the primary event of interest (AKI) from occurring. Abbreviations: CI, confidence interval; KDIGO, Kidney Disease: Improving Global Outcomes; SHR, subdistribution hazard ratio.

Among the 17 of 62 patients who developed AKI during admission, 12 were oliguric or anuric and 4 were non-oliguric (1 patient was missing this information). Competing-risks regression adjusted by study site showed a higher risk of AKI in patients without acetaminophen administration compared to patients receiving acetaminophen (ITT: SHR, 3.0; 95% CI, 1.1 to 8.5; *P* = .034; Figure 3). A total of 2/31 (6%) patients in the acetaminophen group and 6/31 (19%) in the control group received hemodialysis during hospitalization (*P* = .26; Supplementary Table 6). A total of 15/28 (54%) patients admitted with AKI recovered without hemodialysis, of whom 5/15 (33%) were in the control group and 10/15 (67%) in the acetaminophen group (odds ratio, 4.0; 95% CI, 0.7 to 23.9; *P* = .07). Overall case fatality was 4/31 (13%) in the acetaminophen group and 5/31 (16%) in the control group (*P* = .72). Among fatal cases, all had severe malaria on admission (median of 4 severity criteria); 2/9 (22%) had AKI on enrollment and 6/9 (67%) developed AKI after enrollment. Fever and parasite clearance time-to-event analyses found no evidence of a difference between treatment groups by ITT analysis (Supplementary Table 7, Supplementary Figures 6–8). No infecting parasite strain had a mutation in the PfKelch13 gene (a marker for artemisinin resistance).

### Pharmacokinetics and Pharmacodynamics

The population PK properties of acetaminophen were best described by a covariate-free 1-compartment disposition model with 3 transit absorption compartments. The predicted median (IQR) C_MAX_ reached was 16.1 mg/L (14.0 to 20.9 mg/L) and the estimated median (IQR) terminal elimination half-life was 2.79 hours (2.57 to 2.93 hours). Median (IQR) acetaminophen area under the curve (AUC_0__-72h_) in the treatment arm was 386 mg × h × L^-1^ (269 to 496 mg × h × L^-1^) compared to 7 mg × h × L^-1^ (0 to 45 mg × h × L^-1^) in the control arm. Dosing simulations showed that a 6-hourly dose of 1000 mg or 1500 mg resulted in a steady-state mean plasma acetaminophen concentration of 9.21 mg/L and 13.8 mg/L, respectively. A PD mixture model of observed creatinine data, including enrollment creatinine and acetaminophen exposure (AUC_0-72h_) as covariates, best described the data. Higher enrollment creatinine gave a higher probability of a subsequent further deterioration in renal function. The prediction of creatinine change over time was dependent on total acetaminophen exposure where a higher AUC_0-72h_ increased the probability of an improvement in creatinine over the first 72 hours. For example, an enrollment creatinine of 265 μmol/L confers an 83% probability of further deterioration without receiving acetaminophen vs probabilities of 0.02%, 1.2%, or 8.1% with acetaminophen exposures (AUC_0-72h_) of 500, 300, or 200 mg × h × L^-1^, respectively (Table 3). Modeling results showed a positive correlation between acetaminophen exposure and fever clearance time but no correlation with parasite clearance rate. Detailed PK–PD results are provided in the Supplementary Material.

**Table 3.** Pharmacodynamic Prediction of Creatinine Change Over Time Dependent on Baseline Creatinine and Cumulative Acetaminophen Exposure Over 3 Days (AUC_0-72h_)

Baseline Serum Creatinine (mg/dL)	Probability of Belonging to Subpopulation 1 (%)^a^				
	No Acetaminophen	AUC_0-72h_ 100 mg × h/L	AUC_0-72h_ 200 mg × h/L	AUC_0-72h_ 300 mg × h/L	AUC_0-72h_ 500 mg × h/L
1.25 (110.5μmol/L)	10.0	1.46	0.199	0.0266	0.0005
1.50 (132.6μmol/L)	16.0	2.49	0.341	0.0458	0.0008
2.00 (176.8μmol/L)	36.0	7.00	1.00	0.135	0.0024
2.50 (221μmol/L)	62.4	18.2	2.89	0.397	0.0072
3.00 (265.2μmol/L)	83.0	39.6	8.08	1.16	0.0211

Pharmacokinetic-pharmacodynamic (PK–PD) mixture model of observed creatinine described 2 subpopulations, where subpopulation 1 had an increasing creatinine over time and subpopulation 2 had a decreasing creatinine over time. All simulations were based on the developed final model, including enrollment creatinine and acetaminophen AUC_0-72h_ as predictors of the mixture probability. Full details of PK–PD modeling are shown in the Supplementary Material. Conversion from creatinine mg/dL to µmol/L: multiply by 88.4.

Abbreviation: AUC, area under the acetaminophen drug concentration–time curve.

^a^Increasing serum creatinine over time.

### Safety

Median percentage change (IQR) in serum ALT at 72 hours after enrollment was 32% (−9% to 171%) with acetaminophen and −11% (−4% to 57%) in the control group (*P* = .030); for serum AST, this was 0% (−27% to 107%) and −18% (−44% to 1%; *P* = .06; Supplementary Table 6). Analysis of the 2 patients with both an aminotransferase rise >3 times the upper limit of normal (ULN) and a total bilirubin ≥2 times the ULN revealed that the increase in bilirubin was explained by increased unconjugated bilirubin ≥2 times the ULN due to intravascular hemolysis, supported by concomitant elevated lactate dehydrogenase, decreased hematocrit, and blood transfusion requirement. Therefore, no patient met criteria for Hy’s law for hepatotoxicity [19].

## DISCUSSION

This randomized, open-label, controlled trial showed that patients with severe and moderately severe malaria receiving acetaminophen had a larger reduction in serum creatinine and a lower risk of developing AKI compared to control patients not receiving acetaminophen. The beneficial effect of acetaminophen on kidney function was distinctly more pronounced in patients with significant intravascular hemolysis and high concentrations of oxidative stress markers (F_2_-IsoPs and IsoFs). PK–PD modeling showed an acetaminophen exposure–dependent relationship with the improvement in creatinine, which was within the therapeutic dose range. There was a trend toward reduced hemodialysis requirement in the acetaminophen group compared to controls, but the study was not powered to detect an effect toward this endpoint.

The findings support the hypothesis that acetaminophen reduces CFH-mediated oxidative kidney damage and thus would be most beneficial in patients with significant intravascular hemolysis. The results are consistent with recent studies on a heme-mediated oxidative mechanism of AKI and the renoprotective effect of acetaminophen interfering with this mechanism. We recently showed that elevated plasma CFH, F_2_-IsoPs, and IsoFs are associated with in-hospital creatinine rise, hemodialysis requirement, and mortality in patients with severe malaria [10]. Acetaminophen has been shown to reduce toxic ferryl heme to ferric heme in vitro and to decrease plasma F_2_-IsoPs and improve kidney function in a rat model of rhabdomyolysis [11]. A recent randomized trial in septic patients with detectable CFH showed reduced oxidative injury and improved kidney function in patients receiving acetaminophen [14]. While the current study was conducted in patients aged >12 years, these findings may have important implications for the treatment of severe malaria in African children who carry the major burden of this disease and in whom the importance of AKI, hemoglobinuria, and increased plasma CFH is increasingly recognized [20–22]. Further, acetaminophen may also be beneficial in severe *Plasmodium knowlesi* malaria, in which high levels of CFH [23] and a high incidence of AKI have been reported [24], as well as in other diseases characterized by a combination of high CFH and incidence of AKI [4–6, 25].

Studies of acetaminophen in uncomplicated malaria lacking PK–PD analyses have questioned its antipyretic efficacy [26, 27] and suggested deceleration in parasite clearance [26]. PK–PD analysis in the current study shows a dose-dependent effect of acetaminophen on fever clearance time, whereas acetaminophen concentrations were not associated with parasite clearance rates in these artemisinin-sensitive infections. Studies that evaluated acetaminophen in uncomplicated malaria have not reported serious adverse events or hepatotoxicity attributable to acetaminophen [28, 29]. The current study shows that administering the maximum recommended daily dosage of acetaminophen results in a moderate increase in aminotransferases, but no patient met criteria for Hy’s law for hepatotoxicity [19]. The maximum peak acetaminophen concentration of 31.6 mg/L observed in our study is well below the acute toxicity threshold of 150 mg/L used in guidelines as indication for N-acetylcysteine treatment [30]. PK dosing simulations showed that 6-hourly dosing of 1500 mg (6 g/day) achieved therapeutic steady-state acetaminophen concentrations between 10 and 20 mg/L in this patient population, but this exceeds the maximum recommended daily dosage of acetaminophen in adults (4 g/day). The conventional 6-hourly dose of 1000 mg used in this trial yielded a simulated average steady-state concentration of 9.21 mg/L and median AUC_0-72h_ of 644 mg × h × L^-1^ (Supplementary Figure 12). Comparing this cumulative exposure to that generated from the PK–PD model suggests that this conventional regimen has an important renoprotective effect (Table 3).

The study had some limitations. The sample size was relatively small; a larger study is planned. A total of 35/434 (8%) creatinine values were assigned by multiple imputation because of missing samples. However, no imputation was used for the primary endpoint analysis. Patients who received hemodialysis had artificially lowered creatinine values and, according to accepted practice, were assigned an anephric rate of creatinine rise in the analysis [18].

This proof-of-principle study provides evidence that acetaminophen improves kidney function and reduces the risk of developing AKI in severe and moderately severe malaria, particularly in patients with elevated plasma CFH, F_2_-IsoPs, and IsoFs. Since acetaminophen reduces highly oxidative ferryl heme that can be generated with intravascular hemolysis, the findings support the pathophysiological mechanism that CFH-mediated oxidative stress causes kidney injury in severe malaria, a mechanism also relevant for other disease states characterized by intravascular hemolysis. Acetaminophen is inexpensive and widely used, which would facilitate rapid implementation for malaria treatment. A larger trial to evaluate acetaminophen to reduce renal dysfunction in African children with severe malaria is now warranted.

## Supplementary Data

Supplementary materials are available at *Clinical Infectious Diseases* online. Consisting of data provided by the authors to benefit the reader, the posted materials are not copyedited and are the sole responsibility of the authors, so questions or comments should be addressed to the corresponding author.

Supplementary File

## Notes

***Author contribution*.** K. P., R. J. M., L. J. R., and A. M. D. designed the study. K. P., H. W. F. K., M. T. H., S. L., R. J. M., and H. I. performed patient enrollment and data collection. A. G., M. M. U. H., M. S. H., P. K. D., M. A. I., S. A., S. M. J., A. S. M. Z., M. A. S., M. A. H. C., P. C., T. W. Y., M. A. F., and M. A. H. advised on the protocol and supervised clinical care of the participants. K. A. P. conducted the quantification of the cell-free hemoglobin and, with H. W. F. K., interpreted the results. K. S. assisted in coordinating the laboratory field work. K. P., S. J. L., M. M., and A. M. D. conducted the statistical analyses. T. W. and J. T. performed the acetaminophen PK–PD design and analyses. L. J. R. and J. A. O. provided F_2_-IsoP and sofurans quantification and interpretation. K. P. wrote the first draft of the manuscript; A. M. D., G. D. H. T., and N. M. A. edited the initial drafts; and all authors reviewed the final manuscript.

***Acknowledgments*.** We thank the participants who consented to partake in this study, the clinical and laboratory research staff, hospital directors at the study sites, and the following contributors: acetaminophen trial management group: *Mahidol-Oxford Tropical Medicine Research Unit, Bangkok, Thailand*, Arjen Dondorp (chief principal investigator), Katherine Plewes (principal investigator); *Clinical Trials Support Group:* Phaik Yeong Cheah and Zoe Doran; *Data manager:* Thatsanun Ngernseng; *Sample manager:* Mehul Dhorda; *Study statisticians:* Sue J. Lee, Mavuto Mukaka; *PK–PD analyses:* Joel Tarning and Thanaporn Wattanakul; *Bangladesh field site investigators: Chittagong:* Md. Abul Faiz, Md. Amir Hossain, Aniruddha Ghose, Shamsul Alam, Md. Shaiful Haider, Md. Mahtab Uddin Hassan, Prodip Dutta, Selim Md. Jahangir, A.S.M. Zahed, Md. Abdus Sattar, M.A. Hassan Chowdhury, Katherine Plewes, Hugh WF Kingston, Trent Herdman, Stije J. Leopold, Haruhiko Ishioka, Kamolrat Silamut, Benjamas Intharabut, Ketsanee Srinamon, Md. Safiqul Mostafa Choudury, Sanjib Kanti Paul, and Sumon Sarma; *Ramu:* Md. Akhterul Islam, Katherine Plewes, Sukanta Das; *Vanderbilt University School of Medicine:* John A. Oates, L. Jackson Roberts; *Medical monitor:* Arjen Dondorp; *Data safety and monitoring board:* Kasia Stepniewska, Lorenz Von Seidlein, and Piet Kager; *Study monitor:* Tom Peto. We also acknowledge William Zachert for performing quantification of F_2_-IsoPs and IsoFs; Kim A. Piera for conducting quantification of the cell-free hemoglobin; Mallika Imwong and Charlie Woodrow for performing and interpreting the PfKelch13 gene sequencing; and Adeera Levin for input on renal endpoints.

***Disclaimer*.** No funding body or sponsor (University of Oxford) had any role in protocol design, data collection, analysis, interpretation, or writing of the manuscript. The corresponding author had full access to all trial data and assumes final responsibility for the decision to submit for publication. There was no payment from any agency or pharmaceutical company for the writing of this manuscript.

***Financial support*.** This work was supported by the Wellcome Trust of Great Britain (grant 089275/Z/09/Z), the Australian National Health and Medical Research Council (grant605807, and Fellowships to N. M. A. and T. W. Y.), the Bill and Melinda Gates Foundation (grantOPP1134284 to J. T.), and the National Institutes of Health (grantGM15431 to L. J. R. and J. A. O.). K. P. was supported by the Infectious Diseases Society of America Education and Research Foundation and National Foundation for Infectious Diseases Young Investigator Merle A. Sande/Pfizer Fellowship in International Infectious Diseases; and the Clinician Investigator Program at the University of British Columbia, Canada. H. W. F. K. was supported by an Australian Government University Postgraduate Research Scholarship and Prestigious International Research Tuition Scholarship.

***Potential conflicts of interest.*** J. A. O. and L. J. R. have a patent for acetaminophen use pending, neither of whom were involved in protocol development or analysis. All remaining authors declare no competing interests. All authors have submitted the ICMJE Form for Disclosure of Potential Conflicts of Interest. Conflicts that the editors consider relevant to the content of the manuscript have been disclosed.
