# Supplementary material for: Acetaminophen as a Renoprotective Adjunctive Treatment in Patients With Severe and Moderately Severe Falciparum Malaria: A Randomized, Controlled, Open-Label Trial
Source: Clin Infect Dis. 2018 Mar 12;67(7):991–9. doi: 10.1093/cid/ciy213 (PMC6137116; doi:10.1093/cid/ciy213)
Supplement: Supplementary File [file ciy213_suppl_supplementary_file.docx]

**Web extra material**

**Supplementary Appendix:** This appendix has been provided by the authors to communicate additional detail of their work.

**Acetaminophen as a renoprotective adjunctive treatment in patients with severe and moderately severe falciparum malaria: a randomized, controlled, open-label trial**

Katherine Plewes, Hugh W.F. Kingston, Aniruddha Ghose, Thanaporn Wattanakul, Md. Mahtab Uddin Hassan, Md. Shafiul Haider, Prodip K. Dutta, Md. Akhterul Islam, Shamsul Alam, Selim Md. Jahangir, A.S.M. Zahed, Md. Abdus Sattar, M.A. Hassan Chowdhury, M. Trent Herdman, Stije J. Leopold, Haruhiko Ishioka, Kim A. Piera, Prakaykaew Charunwatthana, Kamolrat Silamut, Tsin W. Yeo, Sue J. Lee, Mavuto Mukaka, Richard J. Maude, Gareth D.H. Turner, Md. Abul Faiz, Joel Tarning, John A. Oates, Nicholas M. Anstey, Nicholas J. White, Nicholas P.J. Day, Md. Amir Hossain, L. Jackson Roberts II, Arjen M. Dondorp

Supplementary Material Table of Contents

[METHODS 3](#_Toc507517493)

[RESULTS 6](#_Toc507517494)

[SUPPLEMENTARY TABLES 9](#_Toc507517495)

[SUPPLEMENTARY FIGURES 16](#_Toc507517496)

# **METHODS**

**Trial Design**

The design was a multicenter, open-label, randomized controlled trial at two hospitals in Chittagong Division, Bangladesh. Chittagong Medical College Hospital (CMCH) is a 1000-bed tertiary care referral hospital with basic facilities for intensive care and hemodialysis, and Ramu Upazilla Health Complex is a 31-bed primary care facility with capacity for only blood transfusion. As CMCH is the nearest facility with renal replacement facilities, patients enrolled at the Ramu site requiring dialysis were transferred to CMCH.

**Study Participants**

Eligibility criteria was broadened to include moderately severe malaria after the first year of recruitment (July to September 2012) due to decreased incidence of severe malaria in Bangladesh. Comorbid conditions were determined by patient medical history and medication history review at enrollment. Thus, undiagnosed pre-existing medical conditions may not have been accounted for. Due to resource limitations, hemoglobin A_1_C was not performed to assess for underlying diabetes in patients with significantly elevated random blood glucose at enrollment.

**Laboratory Methods**

Tympanic temperature was recorded as the average of left and right ears; urine color was assessed using a standard urine color chart (Supplementary Figure 1) with hemoglobinuria defined as dark urine with a colorimetric score of ≥6 and urine dipstick positive for hemoglobin with <5 red blood cells per high powered field on urine sediment microscopy. Glucose-6-phosphate dehydrogenase (G6PD) was assessed qualitatively on enrollment with a rapid fluorescent spot test (R&D Diagnostics, Greece). Parasitemia was assessed 6-hourly from thick and thin smears until parasite clearance, defined as two consecutive negative thick smears per 500 white blood cells. Total parasite burden was estimated by plasma *Plasmodium falciparum* histidine rich protein 2 (*Pf*HRP2) using commercial sandwich ELISA (Celisa, Cellabs performed in Bangkok).

In the acetaminophen group, plasma EDTA samples for acetaminophen concentration analysis were collected prior to each dose plus dense sampling after the first (0 hour) and last (72 hour) dose with the following sampling schedule: 0, 0.5, 1.5, 2.5, 4, 6, 12, 18, 24, 30, 36, 42, 48, 54, 60, 66, 72, 72.5, 73.5, 74.5, 76, 78, and 84 hours. Patients in the control group had samples collected 6-hourly for 72 hours to assess unplanned acetaminophen intake. Acetaminophen concentrations were measured using a validated liquid chromatography-tandem mass spectrometry method at the Pharmacokinetics Laboratory at the Chinese University of Hong Kong (Kam RKT *et al.,* submitted). Quality control samples at three levels were analyzed with clinical samples to ensure accuracy and precision through acetaminophen drug measurements (relative bias <15%). Analyses were conducted using Stata (version 14), graphs and individual areas under the acetaminophen drug concentration-time curves (AUCs) were calculated from observed concentrations using the trapezoidal rule in GraphPad version7 (GraphPad Software, USA). Genotyping of the PfKelch13 marker for artemisinin resistance was performed from EDTA samples as described previously [1].

**Population Pharmacokinetic Analysis**

Acetaminophen plasma concentrations were transformed into their natural logarithms and modeled using NONMEM, version 7.3 (Icon Development Solution, Ellicott City, MD). Model diagnostics and automation were performed using Xpose version 4.0 [2], Pirana [3], and Pearl-speaks-NONMEM (PsN; version 3.6) [4]. The first-order conditional estimation method with interaction was used throughout the model development. The difference in objective function value (ΔOFV; calculated by NONMEM as proportional to -2 × the log-likelihood of data) was used as a statistical criterion for discrimination of hierarchical models. ΔOFV of >3.84 and >10.83 were considered statistically significant at p-values of <0.05 and <0.001, respectively, with one degree of freedom difference. Goodness-of-fit and simulation-based diagnostics were used for assessing the descriptive and predictive performances of the model. The data below the limit of quantification were omitted from the pharmacokinetic analysis.

One-, two-, and three-compartment disposition models were evaluated to describe the pharmacokinetic properties of acetaminophen. Different absorption models were investigated to describe the absorption process, including zero-order absorption, first-order absorption with and without lag time, and a flexible transit-absorption model [5]. For patients who had measurable pre-dose concentrations of acetaminophen, baseline estimation was implemented for these patients. Inter-individual variability was introduced exponentially; , where is the individual “ *i* ” parameter estimate, is the population mean parameter estimate, and is the inter-individual variability with zero mean and variance ω^2^. Additionally, the inter-occasion variability was introduced to absorption parameters to describe the variability between dose occasions; $\theta_{ij}=\theta\cdot e^{\eta_{i,\theta}+\kappa_{j,\theta}}$where $\kappa_{j,\theta}$ is the between-occasion variability of the pharmacokinetic parameter $\theta$at the *j’th* dosing occasion. Variability components with an estimated coefficient of variation (%CV) of less than 1% were fixed to zero. The residual unexplained variability was assumed to be additive on a logarithmic scale, essentially equivalent to an exponential error on an arithmetic scale.

Body weight was evaluated as an allometric function on all clearance and volume of distribution parameters using; for all clearance parameters and for all volume of distribution parameters, where BW_i_ represents individual body weight and BW*_median_* represents median body weight of the study population. The influences of demographic patient characteristics were investigated using a stepwise forward inclusion (p-value <0.05) and stepwise backward deletion (p*-*value <0.001) approach. The covariates assessed in the study included: age, INR, AST, ALT, serum creatinine, parasite count, total bilirubin, direct bilirubin, indirect bilirubin, creatinine clearance, hemoglobin, parasitemia, *Pf*HRP2, gender, hemodialysis, severity of malaria disease, and route of acetaminophen administration. Due to the high correlation between disease severity and route of administration in this study, these two variables were investigated also using a full covariate approach to evaluate their impact on acetaminophen pharmacokinetic parameters. Disease severity was included as a categorical covariate on apparent clearance, apparent volume of distribution, and mean transit time. Route of acetaminophen administration was included as a categorical covariate on relative bioavailability and mean transit time. These two full covariate models were bootstrapped (n = 1,000) to investigate the impact of disease severity and route of administration. A change in parameter estimates of more than 25% were deemed to have clinical relevance.

Bootstrapping (n=1,000) was used to assess the robustness of pharmacokinetic parameter estimates from the final model, and to compute relative standard errors and nonparametric confidence intervals for population mean estimates. Numerical and visual predictive checks (n=2,000) were used to evaluate the predictive performance of the final model.

Final pharmacokinetic population parameter estimates from NONMEM were used to simulate different dosing scenarios in Berkeley Madonna [6]. The therapeutic target level of acetaminophen was assumed to be 10-20 mg/L as previously proposed [7, 8]. Different dosage regimens were investigated based on a maximum dose of 4 g acetaminophen per day and the available acetaminophen products (i.e. 500 mg oral tablet). Additionally, individual pharmacokinetic parameter estimates from the final model were imputed directly into the pharmacodynamic model in order to quantify and characterize the drug-dependent pharmacodynamic effects of acetaminophen.

**Pharmacodynamic Analysis**

*Effect of acetaminophen on parasite clearance half-life*

The effect of acetaminophen on parasite clearance half-life was assessed using a pharmacodynamic model that estimated baseline parasite biomass and first-order parasite clearance rate with inter-individual variability on both parameters. To investigate the relationship between acetaminophen concentration and the relative change in parasite clearance rate, individually predicted acetaminophen concentrations were calculated from the final pharmacokinetic model and implemented as a covariate on the parasite clearance rate using a linear relationship and the maximum effect (E_max_) model.

*Effect of acetaminophen on fever clearance time*

A time-to-event (TTE) model was implemented to describe the time to fever clearance. Fever clearance time A (FCT–A: defined as the time to first temperature below 37.5 °C), and fever clearance time B (FCT–B: defined as the time to temperature below 37.5 °C for 24 hours), were coded as events. Patients who died before achieving an event were censored at the time of death.

The TTE models were performed by using the Laplacian estimation with interaction in NONMEM. Data from patients in the control group and treatment group were modelled simultaneously. A constant-hazard model and Weibull distribution hazard model were evaluated. Predicted acetaminophen concentrations were introduced to the baseline hazard (rate of achieving an event) with an E_max_ model: $\text{hz}\left( \text{t} \right)\text{ = }\text{BASE}\text{×}\left( \text{1+}\frac{\text{C}_{\text{p}}}{\text{C}_{\text{p}}\text{ + }\text{EC}_{50}} \right)$, where hz(t) is the hazard at time t, BASE is the constant baseline hazard function, C_p_ is the predicted acetaminophen concentration at time t and EC_50_ is the concentration for a 50% increase of hazard from the baseline value. The survival was calculated by S(t) = exp(-H(t)), where S(t) is the survival at time t and H(t) is the cumulative hazard to time t. The survival calculated here represents the proportion of the patients who did achieve the event. Additionally, the difference of hazard function between control group and treatment group was also evaluated by adding treatment group as a covariate on the hazard function. The pharmacodynamic models were compared by using the difference in objective function value, as described in the pharmacokinetic analysis. The final pharmacodynamic model was evaluated by a Kaplan-Meier visual predictive check (n=1,000), where the observed time to achieve the event (FCT–A, and FCT–B) was overlaid with the 95% prediction interval of the simulated time to achieve the event.

*Effect of acetaminophen on serum creatinine*

A mixture model was implemented to describe the two separate subpopulations of serum creatinine change over time. The physiological relevant covariates including baseline serum creatinine, baseline plasma cell free hemoglobin, treatment arm, and acetaminophen AUC_0-72h_ were evaluated as covariates on the mixture probability using the following equation: $\text{PMIX= }\text{θ}_{\text{PMIX}}\text{×}\left( \text{1+}\text{θ}_{\text{COV-PMIX}}\text{×COV} \right)$.

The probability of belonging to subpopulation 1 (P1: increasing creatinine over time) or subpopulation 2 (P2: decreasing creatinine over time) was calculated using the following equations: P(1) = 1/(1+e^PMIX^) and P(2) = 1-P(1). Different functions were examined to describe the change of serum creatinine over time in each subgroup. The effect of acetaminophen concentrations on the slope of serum creatinine was also investigated using an E_max_ function.

**Statistical Analysis**

A per-protocol (PP) analysis of the secondary outcomes was performed, as presented in the Supplementary Material. In the acetaminophen arm (n=26), 5 patients were excluded for the PP analysis due to: death before receiving acetaminophen (n=3), death within 72 hours (n=1) and withdrawn consent (n=1). In the control arm (no acetaminophen, n=26), 5 patients were excluded for the PP analysis due to: death within 72 hours (n=4) and withdrawn consent (n=1).

# **RESULTS**

**Outcomes**

Mortality was reported as a Serious Adverse Event (SAE) to the medical monitor within 24 hours of death. It was adjudicated that there was no relationship between any patient death and the study drug or study procedures. All patients who died had severe malaria and coma. Death was attributed to severe malaria complicated by multiorgan failure in seven (78%) of the patients where four patients (44%) likely had concomitant bacterial sepsis. Unfortunately, microbiologic confirmation was not possible in these study settings.

Median fever clearance time A (FCT–A) was significantly shorter in patients given acetaminophen compared to the control group only in the per-protocol analysis (ITT: p=0.11; PP: p=0.045) (Supplementary Figure 5; Supplementary Table 7). There was a trend to shorter median FCT–B in patients given acetaminophen compared to controls (ITT: p=0.050; PP: p=0.056) (Supplementary Figure 6; Supplementary Table 7). Median parasite clearance time and parasite half-life were similar in both arms (p=0.10; p=0.12) (Supplementary Figure 7; Supplementary Table 7).

**Pharmacokinetics**

A total of 487 acetaminophen plasma concentration from 28 patients were used in pharmacokinetic analysis. A transit absorption model with fixed number of transit compartments (n = 3) described the absorption phase of acetaminophen optimally compared to a first-order absorption model (ΔOFV = -5.80). The absorption rate constant (k_a_) was set to equal the transit-compartment rate constant (k_tr_) without alteration of model fit or OFV, compared to when estimating k_a_ and k_tr_ separately (ΔOFV = -1.57). Introducing inter-occasion variability on bioavailability to describe random variability on different dosing occasions improved the model fit (∆OFV = -12.0).

Body weight implemented as an allometric covariate on all clearance and distribution parameters did not improve the model fit (ΔOFV = 4.23) and was excluded from the model. The stepwise covariate evaluation included age, gender, ALT, AST, INR, bilirubin (total, indirect and direct), creatinine, creatinine clearance, hemoglobin, parasitemia, *Pf*HRP2, severity, hemodialysis and route of administration. The results during stepwise forward inclusion analysis (p-value <0.05) indicated that female patients had 27.9% lower apparent volume of distribution compared to male patients and the bioavailability was increased by 44.8% in severe patients. However, these two covariates were not retained in the more parsimonious backward elimination analysis (p-value <0.001). The results from the full covariate approach suggested that patients with severe malaria had 26.8% (95%CI, -44.8 to 7.60) lower acetaminophen clearance, equivalent volume of distribution apparent volume of distribution 4.42% (95%CI, -39.2 to 57.9) and decreased mean transit time by 25.0% (95%CI, -56.7 to 42.5) compared to moderately severe patients (Supplementary Figure 8A). The effect of route of administration was investigated using the same approach on absorption parameters. Patients who received acetaminophen syrup by nasogastric tube had 35.9% (95%CI, 2.35 to 65.9) higher bioavailability and 7.94% (95%CI, -37.1 to 47.0) lower mean transit time compared with patients who received oral acetaminophen tablets (Supplementary Figure 8B). Thus, only route of administration showed a statistically significant and clinically important impact on relative bioavailability when using the full covariate approach. However, the effects of route of administration and disease severity were broad and mostly spanned across zero, suggesting that there was insufficient information to conclude these covariate relationships. This was in agreement with the result from the stepwise covariate analysis in which severe patients showed a non-significant trend of higher bioavailability.

Goodness-of-fit diagnostics of the final pharmacokinetic model showed accurate and precise predictions of acetaminophen concentration (Supplementary Figure 9). A small number of concentrations (24/511, 4.70%) were below the level of detection and therefore omitted from the pharmacokinetic analysis. The visual predictive check (n=2000) showed acceptable predictive power of the model (Supplementary Figure 10). The numerical predictive check resulted in 3.90% (95%CI, 2.67 to 7.60%) and 1.44% (95%CI, 2.67 to 7.80%) of acetaminophen concentrations below and above the simulated 90% prediction interval, respectively. Eta shrinkage was less than 35% except for inter-occasion variability of bioavailability (36.6%) and epsilon shrinkage was 8.73%. Parameter estimates from the final acetaminophen pharmacokinetic model are shown in Supplementary Tables 8 and 9.

Dosing simulations showed that severe and moderately severe malaria patients receiving acetaminophen (1,000 mg every 6 hours) reached a mean steady-state concentration (9.21 mg/L) slightly below the therapeutic range (10-20 mg/L) (Supplementary Figure 11A). A loading dose of 1,500 mg followed by 1,000 mg every 6 hours also did not result in a therapeutic steady-state concentration (Supplementary Figure 11B). However, regular dosing of 1,500 mg every 6 hours did achieve a mean steady-state concentration of 13.8 mg/L (Supplementary Figure 11C).

**Pharmacodynamics**

*Effect of acetaminophen on parasite clearance*

The pharmacodynamic model, evaluating the effect of acetaminophen concentration on parasite clearance rate, demonstrated that implementing acetaminophen concentrations (linear or E_max_ function) as a modulator on estimated parasite clearance rate did not improve the model fit (∆OFV = -1.56 and -2.22, respectively). These results suggested that acetaminophen did not affect the parasite clearance rate.

*Effect of acetaminophen on fever clearance time*

Fever clearance time A (FCT–A): A constant hazard model described the time to FCT–A adequately. Implementation of the Weibull distribution hazard model did not improve the model fit (∆OFV = -3.81). Using study group as a covariate on the hazard model suggested that patients in treatment group had a higher rate of FCT–A achievement. However, the covariate did not improve the model fit significantly (∆OFV = -1.98). Fever clearance time B (FCT–B): The Weibull distribution hazard model described the time to FCT–B better than the constant hazard model (∆OFV = -5.03). Using study group as a covariate on the hazard model resulted in a significant model improvement (∆OFV = -11.8) and it showed that patients in the acetaminophen group had a 1.22 times higher rate of achieving FCT–B.

*Effect of acetaminophen on serum creatinine*

A mixture model described the two subpopulations observed in the data; one with increasing (subpopulation 1) and a second with decreasing (subpopulation 2) serum creatinine over time. Adding baseline serum creatinine as a predictor of the probability of belonging to each subpopulation significantly improved the model fit (∆OFV = -31.4). Baseline cell-free hemoglobin, treatment group, and acetaminophen AUC_0-72h_ were evaluated one by one as additional predictors on the mixture probability. Only acetaminophen AUC_0-72h_ improved the model fit significantly (∆OFV = -5.06). The increase in serum creatinine in subpopulation 1 was described by a linear function with a mean slope of 0.0462 h^-1^. In subpopulation 2, the decreasing serum creatinine was described by a one-phase exponential decay function, resulting in an estimated half-life of 49.2 hours and a plateau serum creatinine of 65 µmol/L. Additional acetaminophen concentration-response relationship on the slope of subpopulation 1 and the time to plateau in subpopulation 2 did not show statistical significance (∆OFV = -2.41).

Simulations, using the final model, predicted that patients with baseline serum creatinine of 176.8 μmol/L (2 mg/dL) who did not receive acetaminophen (AUC_0-72h_=0) had 36% mean probability of belonging to subpopulation 1, whereas patients with acetaminophen AUC_0-72h_ of 100, 200, 300, and 500 mg×h×L^-1^ had decreasing mean probabilities of belonging to subpopulation 1 at 7%, 1%, 0.1%, and 0.002%, respectively (Table 3).

# **SUPPLEMENTARY TABLES**

| **Supplementary Table 1. Pre-specified Modified Criteria for Severe Falciparum Malaria [9]** |
| --- |
| Upon hospital admission, asexual parasitemia plus at least one of the following: |
| ⦁ Glasgow coma score < 11 out of 15 |
| ⦁ Generalized convulsions (>2 in 24 hours) |
| ⦁ Acute kidney injury (creatinine >150 μmol/L) |
| ⦁ Jaundice (total bilirubin >2.5 mg/dL + parasitemia >100,000/μL |
| ⦁ Severe anemia (hematocrit <20% + parasitemia >100,000/μL) |
| ⦁ Hyperparasitemia (>5% or > 200,000 parasites/μL) |
| ⦁ Hypoglycemia (glucose <40 mg/dL) |
| ⦁ Venous bicarbonate <15 mmol/L |
| ⦁ Venous lactate >4 mmol/L |
| ⦁ Shock (systolic blood pressure < 80 mmHg with cool extremities) |
| ⦁ Pulmonary edema |
| ⦁ Spontaneous bleeding |
| ⦁ Hemoglobinuria |
| ⦁ Severe prostration (inability to walk or sit without assistance) |
| ⦁ Impaired consciousness (behavioral change, confusion, or drowsiness) |
| Moderately severe malaria was defined as a requirement for inpatient parenteral therapy due to the inability to tolerate oral medication, but no severe malaria criteria. |

| **Supplementary Table 2. Number of Imputed Creatinine Values for Longitudinal Creatinine Analysis** | | | | | | | | | |
| --- | --- | --- | --- | --- | --- | --- | --- | --- | --- |
|  | | Time point (hours) | | | | | | | |
|  | |  | 0 | 12 | 24 | 36 | 48 | 60 | 72 |
| Overall | | N | 62 | 61 | 56 | 54 | 54 | 54 | 54 |
|  |  | Control | 0 | 3 | 1 | 7 | 2 | 3 | 1 |
|  |  | Acetaminophen | 0 | 4 | 0 | 8 | 1 | 4 | 1 |
| CFH ≥ 45,000 ng/mL | | N | 31 | 30 | 25 | 24 | 24 | 24 | 24 |
|  | | Control | 0 | 3 | 1 | 2 | 0 | 0 | 0 |
|  | | Acetaminophen | 0 | 2 | 0 | 3 | 0 | 1 | 0 |
| CFH < 45,000 ng/mL | | N | 31 | 31 | 31 | 30 | 30 | 30 | 30 |
|  | | Control | 0 | 0 | 0 | 5 | 2 | 3 | 1 |
|  | | Acetaminophen | 0 | 2 | 0 | 5 | 1 | 3 | 1 |
| Note: In addition to the complete case analysis, a multiple imputation procedure with five rounds was employed. The number of imputed creatinine observation at each time point is shown. Missing values are due to creatinine values not imputed after time of death. | | | | | | | | | |

| **Supplementary Table 3. Mean Percent Change Imputed Creatinine from Enrollment of Longitudinal Creatinine Concentrations Intention-to-Treat Analysis** | | | | |
| --- | --- | --- | --- | --- |
|  | Acetaminophen | N | Control | N |
| **Mean % change from enrollment** | |  |  |  |
| Creatinine at enrollment (μmol/L) ^a^ | 106 (97–169) | 31 | 115 (97–168) | 31 |
| % peak change creatinine | 2 (33) | 31 | 18 (52) | 30 |
| % Δ creatinine 12 hours | -5 (17) | 31 | -5 (18) | 30 |
| % Δ creatinine 24 hours | -5 (32) | 28 | -6(24) | 28 |
| % Δ creatinine 36 hours | -18 (18) | 27 | -3 (36) | 27 |
| % Δ creatinine 48 hours | -18 (20) | 27 | 1 (42) | 27 |
| % Δ creatinine 60 hours | -22 (23) | 27 | 3 (52) | 27 |
| % Δ creatinine 72 hours | -22 (27) | 27 | 7 (60) | 27 |
| **Mean % change CFH ≥ 45,000 ng/mL** | |  |  |  |
| Creatinine at enrollment (μmol/L) ^a^ | 141 (97–221) | 13 | 136 (97–239) | 18 |
| % peak change creatinine | -6 (14) | 13 | 24 (52) | 17 |
| % Δ creatinine 12 hours | -6 (14) | 13 | -1 (20) | 17 |
| % Δ creatinine 24 hours | -18 (9) | 10 | -1 (27) | 15 |
| % Δ creatinine 36 hours | -27 (12) | 10 | 3 (36) | 14 |
| % Δ creatinine 48 hours | -28 (12) | 10 | 7 (45) | 14 |
| % Δ creatinine 60 hours | -34 (11) | 10 | 9 (55) | 14 |
| % Δ creatinine 72 hours | -35 (12) | 10 | 14 (64) | 14 |
| **Mean % change CFH < 45,000 ng/mL** | |  |  |  |
| Creatinine at enrollment (μmol/L) ^a^ | 106 (97–115) | 18 | 106 (97–124) | 13 |
| % peak change creatinine | 9 (40) | 18 | 10 (54) | 13 |
| % Δ creatinine 12 hours | -4 (19) | 18 | -11 (12) | 13 |
| % Δ creatinine 24 hours | 3 (37) | 18 | -11 (20) | 13 |
| % Δ creatinine 36 hours | -12 (19) | 17 | -9 (37) | 13 |
| % Δ creatinine 48 hours | -13 (22) | 17 | -6 (40) | 13 |
| % Δ creatinine 60 hours | -15 (26) | 17 | -4 (49) | 13 |
| % Δ creatinine 72 hours | -14 (30) | 17 | 1 (58) | 13 |
| Data are means (SD) unless otherwise indicated by ^a^median (IQR). Approximately 8% of total creatinine time points were missing sample collections (median, range; 1 (0,5). Multiple imputation procedure with five rounds was employed. The number and time point of each imputed value is detailed in Supplementary Table 2.  Abbreviations: CFH, cell-free hemoglobin. | | | | |

| **Supplementary Table 4. Mean Percent Change Creatinine from Enrollment of Longitudinal Creatinine Concentrations Intention-to-Treat Complete Case Analysis** | | | | | |
| --- | --- | --- | --- | --- | --- |
|  | Acetaminophen | N | Control | N |
| **Mean % change from enrollment** |  |  |  |  |
| Creatinine at enrollment (μmol/L) ^a^ | 106 (97–169) | 31 | 115 (97–168) | 31 |
| % peak change creatinine | 1 (33) | 30 | 19 (54) | 28 |
| % Δ creatinine 12 hours | -6 (18) | 27 | -5 (18) | 27 |
| % Δ creatinine 24 hours | -5 (32) | 28 | -6 (25) | 27 |
| % Δ creatinine 36 hours | -17 (18) | 19 | -3 (36) | 20 |
| % Δ creatinine 48 hours | -18 (21) | 26 | -2 (39) | 25 |
| % Δ creatinine 60 hours | -22 (24) | 23 | 0 (48) | 24 |
| % Δ creatinine 72 hours | -23 (27) | 26 | 9 (61) | 26 |
| **Mean % change CFH ≥ 45,000 ng/mL** | |  |  |  |
| Creatinine at enrollment (μmol/L) ^a^ | 141 (97–221) | 13 | 136 (97–239) | 18 |
| % peak change creatinine | -8 (13) | 12 | 27 (55) | 15 |
| % Δ creatinine 12 hours | -8 (13) | 11 | 1 (22) | 14 |
| % Δ creatinine 24 hours | -18 (9) | 10 | -1 (28) | 14 |
| % Δ creatinine 36 hours | -23 (11) | 7 | 5 (39) | 12 |
| % Δ creatinine 48 hours | -28 (12) | 10 | 7 (45) | 14 |
| % Δ creatinine 60 hours | -32 (10) | 9 | 9 (55) | 14 |
| % Δ creatinine 72 hours | -35 (12) | 10 | 14 (64) | 14 |
| **Mean % change CFH < 45,000 ng/mL** | |  |  |  |
| Creatinine at enrollment (μmol/L) ^a^ | 106 (97–115) | 18 | 106 (97–124) | 13 |
| % peak change creatinine | 8 (41) | 18 | 10 (54) | 13 |
| % Δ creatinine 12 hours | -4 (20) | 16 | -11 (12) | 13 |
| % Δ creatinine 24 hours | 3 (37) | 18 | -11 (20) | 13 |
| % Δ creatinine 36 hours | -13 (21) | 12 | -15 (29) | 8 |
| % Δ creatinine 48 hours | -12 (23) | 16 | -14 (28) | 11 |
| % Δ creatinine 60 hours | -16 (28) | 14 | -13 (36) | 10 |
| % Δ creatinine 72 hours | -15 (31) | 16 | 4 (59) | 12 |
| Data are means (SD) unless otherwise indicated by ^a^median (IQR).  Abbreviations: CFH, cell-free hemoglobin. | | | | | |

| **Supplementary Table 5. Mixed Effects Models of Percent Change in Creatinine over Time** | | | | |
| --- | --- | --- | --- | --- |
| Variable | Study group | Coefficient | 95% CI | *P* |
| **MODEL 1** |  |  |  |  |
| **Entire cohort** |  |  |  |  |
| Treatment group | Acetaminophen | -3.4 | -4.7 to -2.1 | < 0.001 |
| Time |  | 1.7 | 0.4 to 3.0 | 0.011 |
| Treatment × time |  | -5.1 | -6.9 to -3.2 | < 0.001 |
| **CFH ≥ 45,000 ng/mL** |  |  |  |  |
| Treatment group | Acetaminophen | -5.7 | -7.7 to -3.7 | < 0.001 |
| Time |  | 2.6 | 0.9 to 4.3 | 0.003 |
| Treatment × time |  | -8.3 | -10.9 to -5.6 | < 0.001 |
|  |  |  |  |  |
| **CFH < 45,000 ng/mL** |  |  |  |  |
| Treatment group | Acetaminophen | -2.1 | -3.7 to -0.4 | 0.01 |
| Time |  | 0.8 | -1.2 to 2.7 | 0.44 |
| Treatment × time |  | -2.8 | -5.4 to -0.3 | 0.031 |
| **MODEL 2** |  |  |  |  |
| **Entire cohort** |  |  |  |  |
| Treatment group | Acetaminophen | 111.7 | 23.6 to 200 | 0.013 |
| Time |  | 1.7 | 0.43 to 3.0 | 0.009 |
| Treatment × time |  | -5.2 | -7.0 to -3.3 | < 0.001 |
| Cell-free hemoglobin |  | 5.9 | 0.3 to 11.5 | 0.038 |
| Cell-free hemoglobin × treatment |  | -9.8 | -17.8 to -1.8 | 0.016 |
| Model 1: Mixed effects model with a treatment×time interaction term assessing if the effect of acetaminophen on proportional creatinine change is modified by time.  Model 2: Mixed effects model with the addition of a cell-free hemoglobin×treatment group interaction term assessing if the dependence of proportional creatinine change on acetaminophen depends on the concentration of cell-free hemoglobin at enrollment.  Abbreviations: CFH, cell-free hemoglobin; CI, confidence intervals. | | | | |

| **Supplementary Table 6. Outcomes by Treatment Group** | | | | | | |
| --- | --- | --- | --- | --- | --- | --- |
|  | Acetaminophen | N | Control | N | *P* |
| **Renal** |  |  |  |  |  |
| Recovery of enrollment AKI without RRT | 10 (71) | 14 | 5 (36) | 14 | 0.06 |
| Hemodialysis | 2 (6) | 31 | 6 (19) | 31 | 0.26 |
| Time to hemodialysis, (hours)^β^ | 7 (6–8) | 2 | 4 (3–5) | 6 | 0.13 |
| Number of dialysis cycles | 4 (1–6) | 2 | 5 (3–6) | 6 | 0.61 |
| **Acetaminophen** |  |  |  |  |  |
| AUC_0–72 hr_ (mg×h/l)^#^ | 386 (269–496) | 28 | 7 (0–45) | 28 | **<0.001** |
| **Hepatotoxicity** |  |  |  |  |  |
| ALT_0 hr_ (U/L) | 19 (13–28) | 31 | 23 (18–37) | 31 | 0.11 |
| ALT_72 hr_ (U/L) | 31 (15–61) | 26 | 23 (16–38) | 25 | 0.28 |
| Δ ALT_0–72 hr_ (%) | 32 (-9–171) | 26 | -11 (-34–57) | 25 | **0.030** |
| ALT_72hr_ >3-fold ULN + total bilirubin >2×ULN^¶^ | 1 (6)* | 18 | 0 (0) | 21 | 0.46 |
| ALT >3-fold ULN + indirect bilirubin ≥2×ULN | 1 (6)* | 18 | 0 (0) | 21 | 0.46 |
| AST_0 hr_ (U/L) | 42 (29–81) | 31 | 52 (29–112) | 31 | 0.32 |
| AST_72 hr_ (U/L) | 60 (20–81) | 21 | 36 (25–57) | 20 | 0.25 |
| Δ AST_0–72 hr_ (%) | 0 (-27–107) | 21 | -18 (-44–1) | 20 | 0.06 |
| Total bilirubin_0 hr_ (mg/dL)^a^ | 1.5 (1.0–2.2) | 31 | 1.7 (1.2–2.4) | 31 | 0.63 |
| Total bilirubin_72 hr_ (mg/dL)^a^ | 0.8 (0.6–1.2) | 24 | 0.7 (0.5–0.9) | 22 | 0.45 |
| Δ total bilirubin_0–72 hr_ (%) | -33 (-68– -9) | 24 | -55 (-71– -7) | 22 | 0.31 |
| Direct bilirubin_0 hr_ (mg/dL)^a^ | 0.7 (0.4–1.1) | 31 | 0.7 (0.5–1.1) | 31 | 0.83 |
| Direct bilirubin_72 hr_ (mg/dL)^a^ | 0.2 (0.1–0.4) | 24 | 0.2 (0.2–0.3) | 22 | 0.96 |
| Δ direct bilirubin_0–72 hr_ (%) | -50 (-82– -24) | 24 | -66 (-77– -50) | 22 | 0.53 |
| Indirect bilirubin_0 hr_ (mg/dL)^a^ | 0.7 (0.5–0.9) | 31 | 0.8 (0.6–1.2) | 31 | 0.29 |
| Indirect bilirubin_72 hr_ (mg/dL)^a^ | 0.5 (0.4–0.7) | 24 | 0.4 (0.3–0.6) | 22 | 0.40 |
| Δ indirect bilirubin_0–72 hr_ (%) | -29 (-49–33) | 24 | -49 (-73–50) | 22 | 0.43 |
| AST_72 hr_ >3-fold ULN + total bilirubin >2×ULN^¶^ | 2 (18)* | 11 | 0 (0) | 12 | 0.22 |
| AST_72 hr_ >3-fold ULN + indirect bilirubin ≥2×ULN | 2 (18)* | 11 | 0 (0) | 12 | 0.22 |
| **Survival** |  |  |  |  |  |
| Mortality^β^ | 4 (13) | 31 | 5 (16) | 31 | 0.72 |
| Time to death (hours) | 19 (18–24) | 4 | 16 (7–26) | 5 | 0.46 |
| Data are number (%) or median (IQR), unless otherwise indicated by ^a^ geometric mean (95% CI).  Fisher's exact test and Mann Whiney-U were used for evaluating differences between categorical and continuous data. ^a^ T-tests used for evaluating differences between log normal transformed data. ALT and AST upper limit of normal (ULN) for males and females was 50 U/L and 35 U/L, respectively. Indirect bilirubin calculated by total bilirubin minus direct bilirubin.  Abbreviations: AUC, area under the acetaminophen concentration–time curve; AKI, acute kidney injury; ALT, alanine aminotransferase; AST, aspartate aminotransferase. RRT, renal replacement therapy.  ^β^One patient in the control group died after 72 hours.  ^#^Four patients in the control group received acetaminophen (range, 1–3 doses); of whom three had an AUC <100 mg×h/l, and one with an AUC of 292 mg×h/l whose creatinine was normal throughout enrollment.  ¶ Hy's Law requires that (i) a drug causes hepatocellular injury, shown by more frequent 3-fold or greater elevations above the ULN of ALT or AST, than control or placebo, (ii) among those with ALT or AST > than 3×ULN, may show elevation in serum total bilirubin to >2×ULN, without initial findings of cholestasis, and (iii) no other reason explains the combination of ALT/AST and bilirubin increases.  *Assessment of the two patients with both an aminotransferase rise > 3 times the upper limit of normal and a total bilirubin >2 ULN revealed that the increase in total bilirubin was explained by increased unconjugated bilirubin >2 ULN due to intravascular hemolysis, supported by concomitant elevated LDH, decreased hematocrit, and blood transfusion requirement. | | | | | | | |

| **Supplementary Table 7. Secondary Outcomes by Treatment group** | | | | | | |
| --- | --- | --- | --- | --- | --- | --- |
|  | Acetaminophen | N | Control | N | *P* |
| **Parasite clearance** |  |  |  |  |  |
| Parasite clearance time: ITT (hours) | 54 (40­–63) | 30 | 42 (36–55) | 31 | 0.10 ^β^ |
| Parasite clearance time: PP (hours) | 54 (48–63) | 25 | 42 (36–55) | 26 | 0.07 ^β^ |
| Parasite half-life: ITT (hours) | 3.1 (2.0–3.7) | 28 | 3.4 (2.9–5.1) | 29 | 0.12 |
| Parasite half-life: PP (hours) | 3.2 (2.0–3.7) | 25 | 3.4 (2.8–5.1) | 26 | 0.24 |
| **Fever clearance** |  |  |  |  |  |
| Fever clearance time A: ITT (hours) | 9.7 (5.8–30.1) | 22 | 18.5 (7.0–37.0) | 24 | 0.11^ζ^ |
| Fever clearance time A: PP (hours) | 7.9 (5.8–25.2) | 19 | 18.5 (7.0–37.0) | 20 | **0.045** ^ζ^ |
| Fever clearance time B: ITT (hours)* | 37.0 (24.7–61.1) | 29 | 60.3 (30.9–86.0) | 30 | 0.050 ^ζ^ |
| Fever clearance time B: PP (hours)* | 31.0 (18.3–44.1) | 25 | 59.8 (30.9–86.0) | 25 | 0.056 ^ζ^ |
| Area above 37.5°C: ITT | 16.1 (3.8–24.6) | 31 | 21.8 (12.0–33.8) | 31 | 0.10 |
| Area above 37.5°C: PP | 18.5 (6.6–24.6) | 26 | 24.3 (14.1–35.5) | 26 | 0.09 |
| Data are median (IQR), unless otherwise indicated.  Fever clearance time A was defined as time to first temperature below 37.5°C; fever clearance time B was defined as time to first temperature that remained below 37.5°C for 24 hours; area above the 37.5°C temperature versus time curve was calculated using the trapezoidal rule.  Wilcoxon-Mann-Whiney test was used to compare area above the curve and parasite half-life. Time to event outcomes were compared using:  ^ζ^ log rank test, or  ^β^Wilcoxon-Breslow-Gehan test if lines crossed.  Abbreviations: ITT, intention-to-treat; PP, per protocol.  *One asplenic patient with a parasite clearance time of 509 hours was excluded from fever clearance time B analyses. | | | | | |

**Supplementary Table 8. Parameter Estimates of the Final Population Pharmacokinetic Model of Acetaminophen in Patients with Severe and Moderately Severe Malaria.**

| Parameter | Population estimates^a^  (%RSE)^b^ | 95%CI^b^ | IIV^a^ [%CV]  (%RSE)^b^ | 95%CI^b^ | IOV^a^ [%CV]  (%RSE)^b^ | 95%CI^b^ |
| --- | --- | --- | --- | --- | --- | --- |
| F | 1 (fixed) | … | 24.8 (27.2) | 10.8–39.1 | 32.8 (24.5) | 12.8–50.6 |
| CL/F (L/h) | 18.1 (7.78) | 15.7–21.2 | … | … | … | … |
| V/F (L) | 73.5 (12.8) | 57.7–95.1 | 17.6 (32.7) | 5.27­–29.4 | … | … |
| MTT (h) | 0.494 (10.3) | 0.393–0.589 | … | … | … | … |
| σ | 0.655 (22.7) | 0.403–0.869 | … | … | … | … |

Abbreviations: F, relative bioavailability; MTT, mean transit absorption time; CL/F, apparent oral elimination clearance; V/F, apparent volume of distribution of the central compartment; σ, variance of the residual variability.

^a^ Population mean values, inter-individual variability (IIV), and inter-occasion variability (IOV) were estimated by NONMEM. The coefficient of variation (%CV) for IIV and IOV were calculated as $\text{100×}\sqrt{\exp\left( \text{estimate} \right)\text{-1}}$.

^b^ The relative standard error (%RSE) was calculated as $\text{100×(}\frac{\text{SD}}{\text{Mean value}}\text{)}$ from the non-parametric bootstrap results (n=1,000). The 95% confidence interval (95%CI) is presented as the 2.5 to 97.5 percentiles of bootstrap estimates.

**Supplementary Table 9. Secondary Parameters of Acetaminophen from the Final Pharmacokinetic Model**

| Secondary parameters | Median (IQR) |
| --- | --- |
| C_MAX_ (mg/L) | 16.1 (14.0–20.9) |
| T_MAX_ (h) | 0.961 (0.949–0.977) |
| T_1/2_ (h) | 2.79 (2.57–2.93) |
| Dose-normalized AUC_0-24_ (mg×h×L^-1^) | 210 (199–297) |

Abbreviations: C_MAX_, maximum concentration; T_MAX_, time to reach maximum concentration; T_1/2_, terminal elimination; Dose normalized AUC_0-24_, area under the concentration-time curve from 0 to 24 hours normalized by 4g of acetaminophen dose.

# **SUPPLEMENTARY FIGURES**


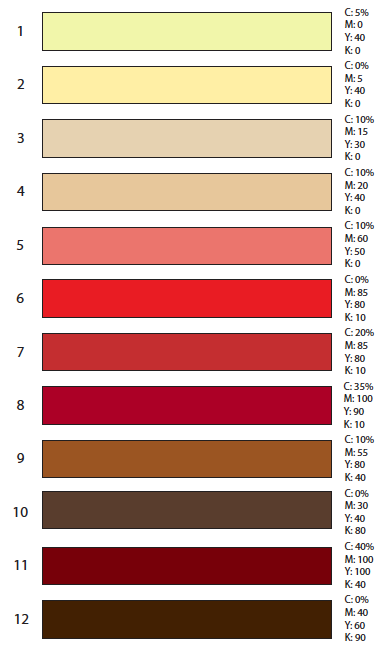


**Supplementary Figure 1.** Urine color scale to detect hemoglobinuria. Color scale was adapted from Hillmen *et al.* [10] to monitor for incidence of hemoglobinuria in falciparum malaria patients. Color scale was created in Photoshop using cyan, magenta, yellow and key (black) (CMYK) color model as listed for chart reproducibility. Hemoglobinuria was prospectively defined as a urine color of 6 or greater. Urine was aliquoted into clear 10 mL collection tubes with a 0.5 cm diameter in order to standardize color assessments between and within patients. Tubes filled with urine were held against a white background in a well-lit room in order to assess the color with reference to the color scale.


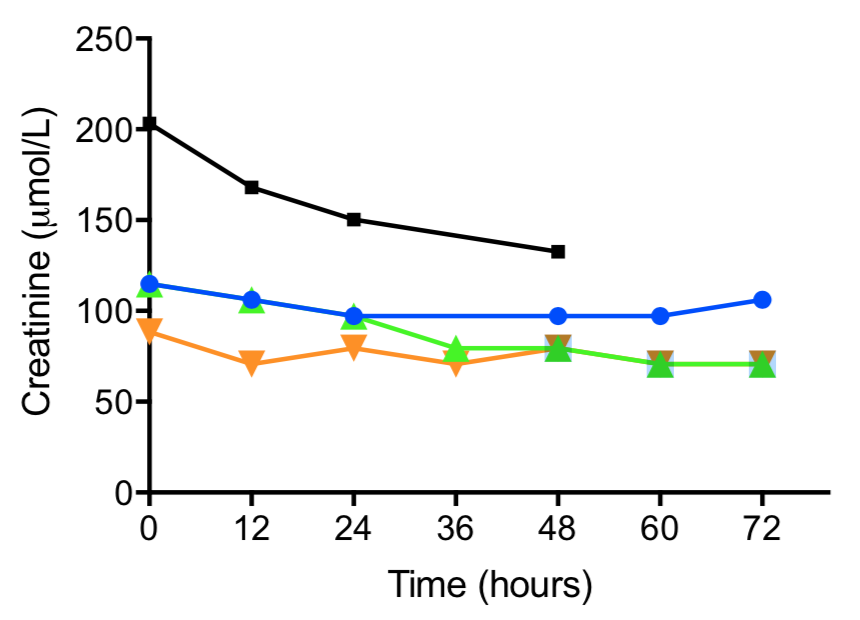


**Supplementary Figure 2.**  Creatinine over time among control group patients who received non-steroidal anti-inflammatories. All patients received one diclofenac (or ibuprofen) suppository except one patient who received 2 doses (blue line). One patient (black line) was withdrawn due to blood draw refusal and did not have creatinine beyond 48 hours.


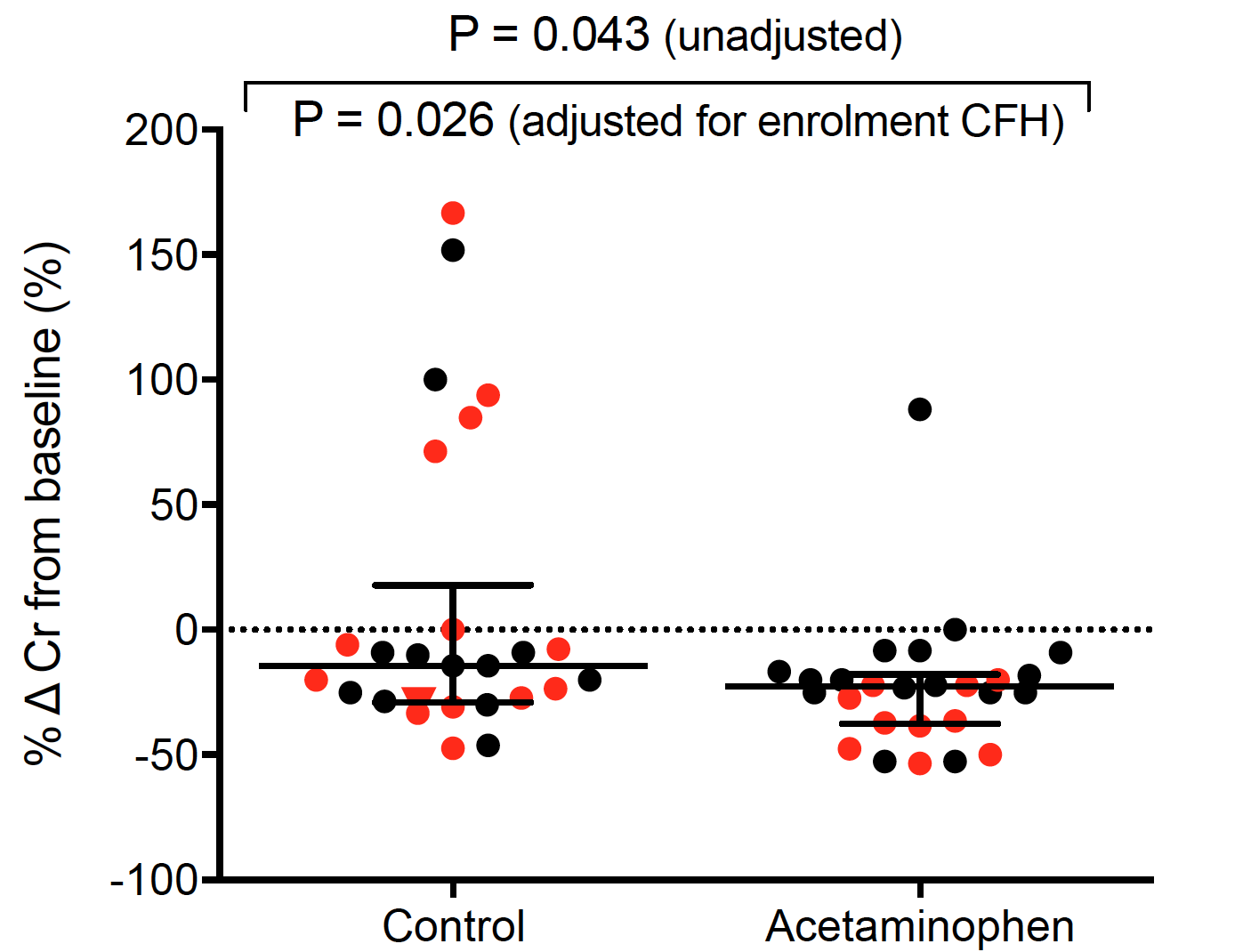


**Supplementary Figure 3.** Acetaminophen effect on 72-hour creatinine. Percentage change in creatinine from enrollment to 72 hours was the primary endpoint. Red and black solid dots represent patients with enrollment plasma CFH ≥ 45,000 ng/mL, and plasma CFH < 45,000 ng/mL, respectively. Red triangle represents a patient in the control group with elevated acetaminophen area under the concentration-time curve. Unadjusted between-group differences were compared with Wilcoxon-Mann-Whitney test (p=0.043). Adjusting for enrollment CFH using linear regression, the 72-hour percent change of creatinine from enrollment was significant (p=0.026; treatment difference: -31%, 95%CI, -58% to -4%). Abbreviations: Cr, creatinine; CFH, cell-free hemoglobin.


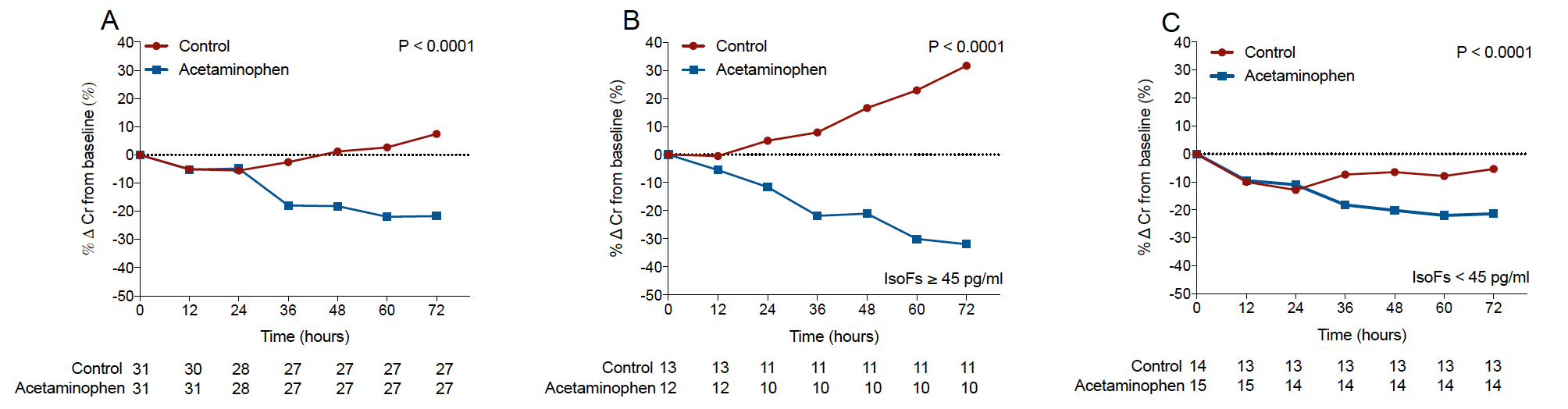


**Supplementary Figure 4.** Effect of acetaminophen on creatinine stratified by median isofuran concentration. Creatinine mean percent change from enrollment at 12, 24, 36, 60, 48, and 72 hours, of: (A) entire cohort, (B-C) patients stratified by level of lipid peroxidation: B: plasma isofurans ≥45 pg/mL, C: plasma isofurans <45 pg/mL. Analyses using mixed effects models, with the maximum likelihood method of estimation. Among those with IsoF ≥45 pg/mL; acetaminophen group coefficient: -5.3 (95%CI, -7.5 to -3.1) versus control group: 5.6 (95%CI, 3.5 to 7.7; p<0.001). Across time points there were missing sample collections (median, range; 1 (0 to 5)), approximately 8% of total creatinine sampling time points. In addition to the complete case analysis, multiple imputation procedure with five rounds was employed. The number of imputed creatinine observations at each time point is detailed in Supplementary Table 2. Frequencies in rows below figures represent number of patients (n) at each time point. P-values represent overall treatment effect of treatment calculated using lincom in STATA.

Abbreviations: Cr, creatinine; IsoFs, isofurans.


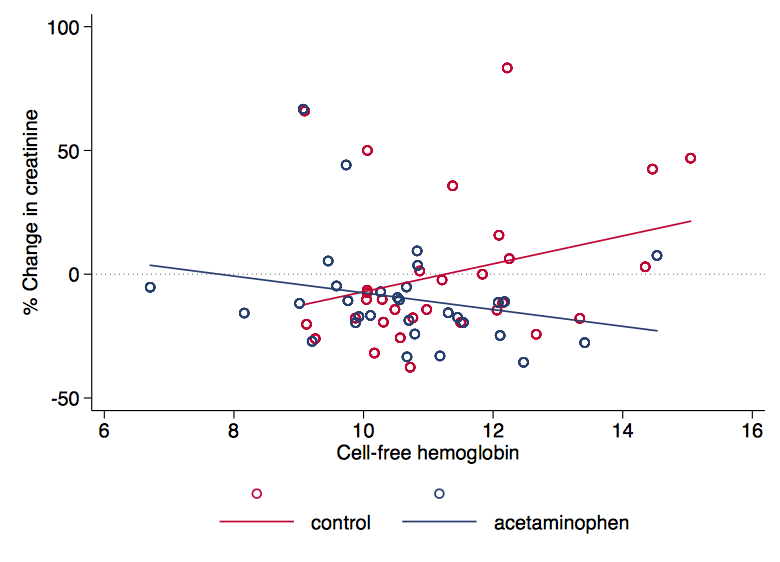


**Supplementary Figure 5.** Mixed effects model of percent change creatinine accounting for cell-free hemoglobin. Interaction terms included cell-free hemoglobin (log transformed) with treatment group and treatment group with time. The effect of acetaminophen on the reduction of serum creatinine depended on enrollment CFH, where higher admission CFH was associated with a more pronounced reduction in creatinine with acetaminophen administration (interaction p-value=0.016).


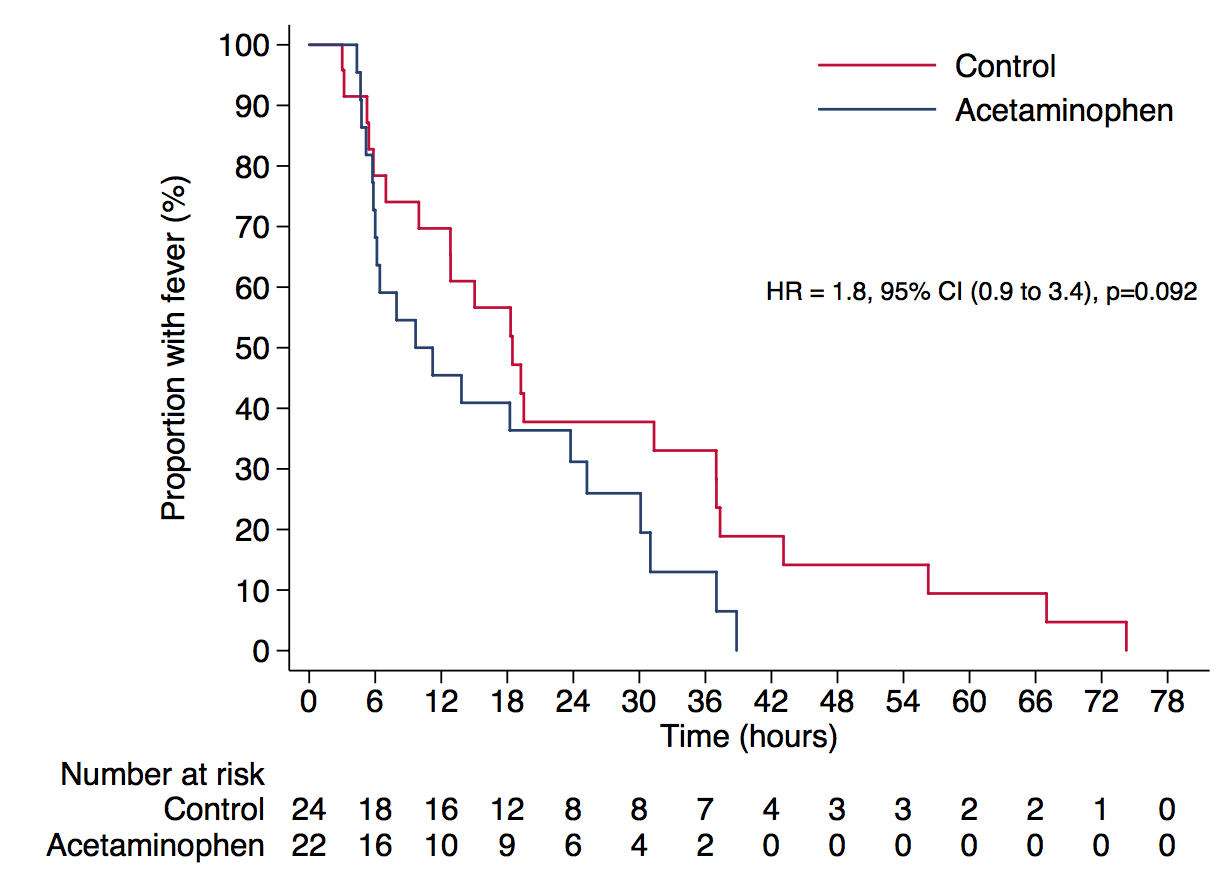


**Supplementary Figure 6.** Kaplan-Meier comparing fever clearance A in patients with severe and moderately severe malaria treated with either acetaminophen or no acetaminophen (control). Fever clearance defined as the time to first aural temperature below 37.5°C from enrollment (FCT–A). Patients were censored at the time of first aural temperature measuring less than 37.5°C or time of death. Sixteen patients were afebrile on admission and not included in this analysis. P value, Cox regression Wilcoxon-Breslow-Gehan test adjusted for baseline temperature; intention-to-treat. Per protocol analysis: HR=2.1, 95%CI, 1.1 to 4.3; p=0.031 adjusted for baseline temperature.


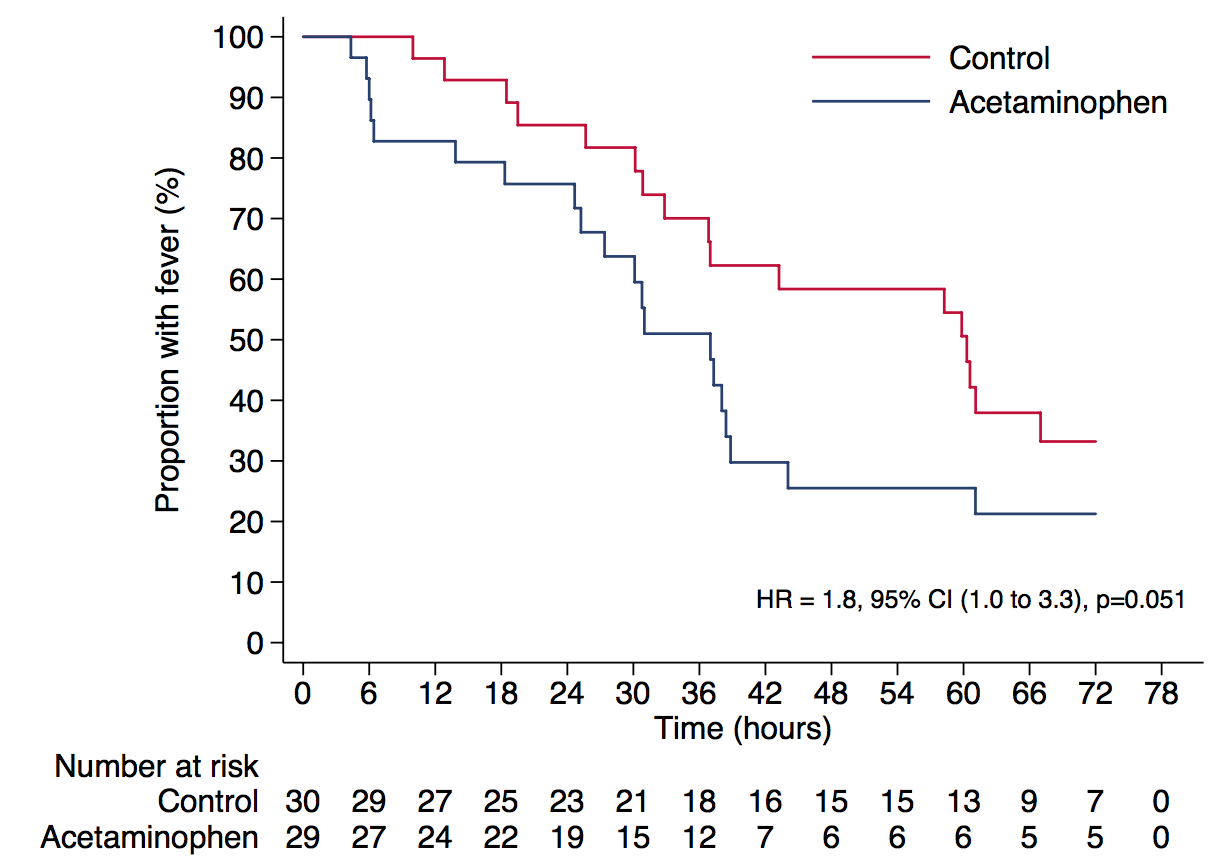


**Supplementary Figure 7.** Kaplan-Meier comparing fever clearance B in patients with severe and moderately severe malaria treated with either acetaminophen or no acetaminophen (control). Fever clearance defined as the time to first aural temperature remaining below 37.5°C for 24 hours since enrollment (FCT–B). Patients were censored at the time of first aural temperature measuring less than 37.5°C for a 24-hour duration or time of death. P value, Wilcoxon-Breslow-Gehan test adjusted for baseline temperature; intention-to-treat. Per protocol analysis: HR=1.8, 95%CI, 1.0 to 3.3; p=0.060. Analysis includes two patients with prolonged fever clearance >300 hours of non-malarial etiology. Excluding two patients who had a FCT-B of greater than 300 hours due to likely concomitant bacterial infection (one patient in each group), the median (IQR) FCT-B was shorter in patient given acetaminophen (31.0 hours (18.3 to 44.1 hours) compared to controls (59.8 hours (30.9 to 85.3 hours); p=0.043).


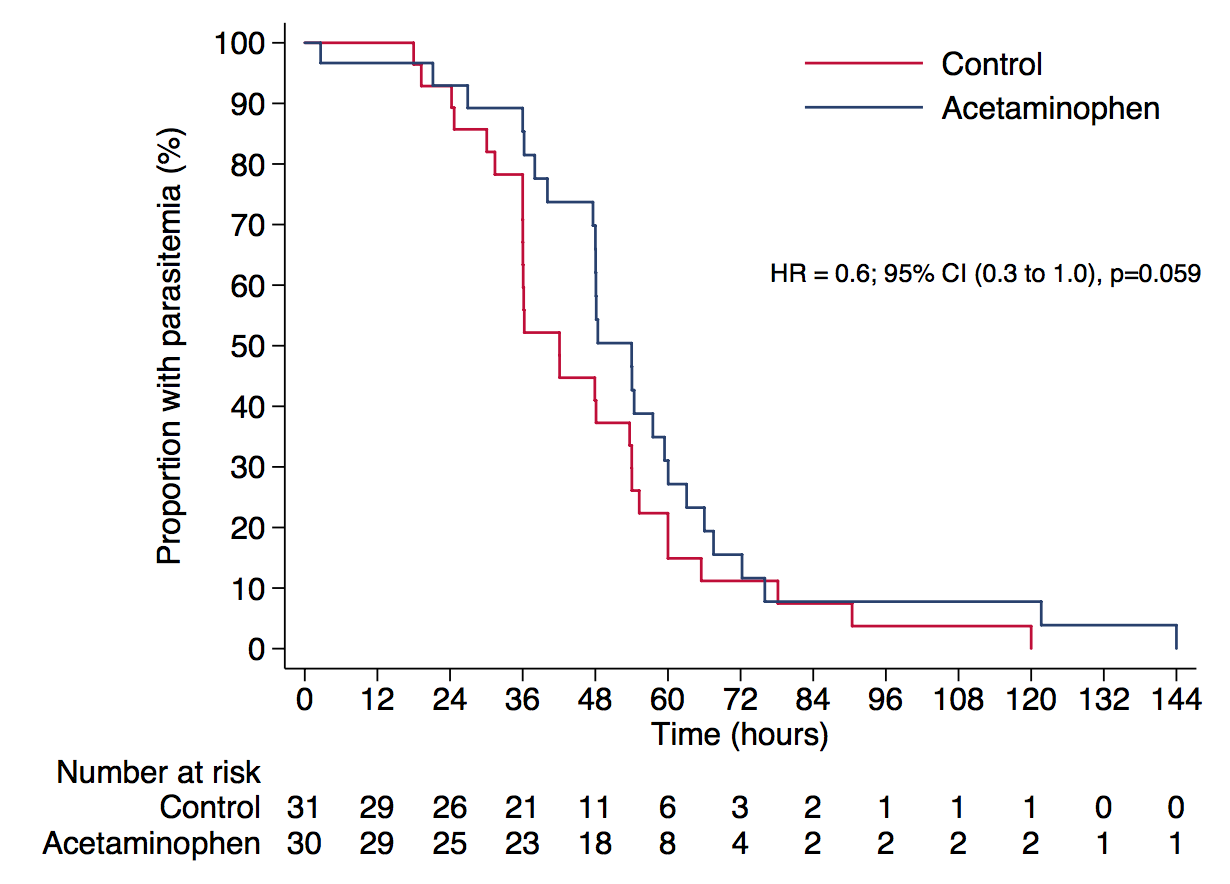


**Supplementary Figure 8.** Kaplan-Meier comparing parasite clearance time in patients with severe and moderately severe malaria treated with either acetaminophen or no acetaminophen (control). Parasite clearance defined as the time from enrollment to the first of two consecutively negative thick smears (per 500 white blood cells). Patients were censored at the time of first negative smear or time of death. P value, Wilcoxon-Breslow-Gehan test adjusted for baseline parasitemia; intention-to-treat. Per protocol analysis: HR=0.6, 95%CI, 0.3 to 1.0; p=0.053. Analysis excludes an asplenic patient in acetaminophen group with PCT > 500 hours [11].

**Supplementary Figure 9.** Effect of severity (A) and route of administration (B) on acetaminophen pharmacokinetic parameters. The shaded areas represent a covariate effect of ±25%, defined as clinically unimportant. Abbreviations: MTT, mean transit absorption time; V/F, apparent volume of distribution of the central compartment; CL/F, apparent oral elimination clearance; F, relative bioavailability.

**Supplementary Figure 10.** Goodness-of-fit diagnostics of final acetaminophen population pharmacokinetic model. Observations are represented by the black circles; solid black line represents the line of identity (zero line), dashed line represents the local polynomial regression. Observed plasma acetaminophen concentrations, population predictions, and individual predictions were log transformed (base 10).


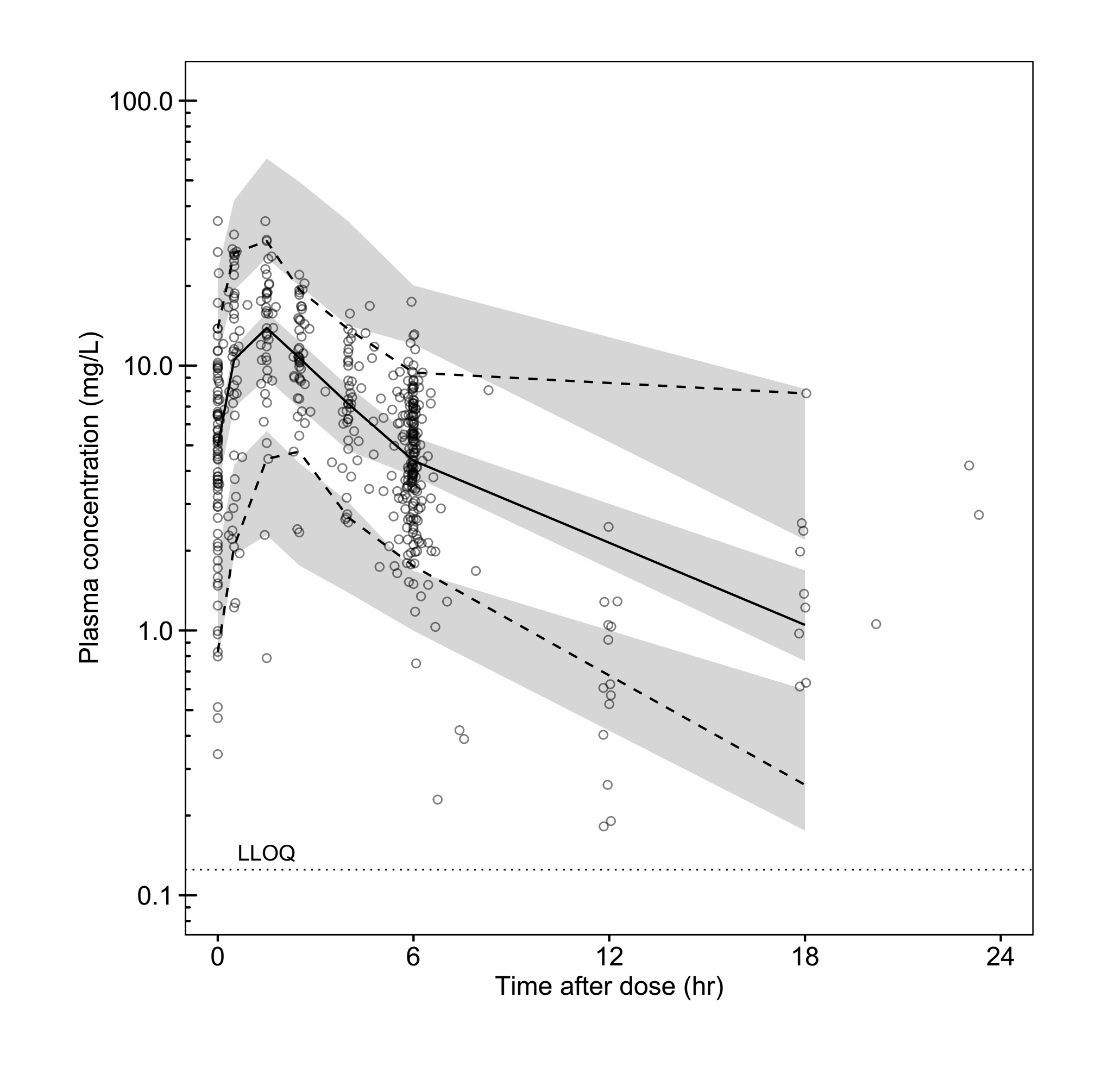


**Supplementary Figure 11.** Prediction-corrected visual predictive check of the final acetaminophen population pharmacokinetic model in patients with severe and moderately severe falciparum malaria. Open circles represent observed data points; solid and dashed lines represent the 5^th^, 50^th^, and 95^th^ percentiles of the observed data; shaded areas represent the 95% confidence intervals of the simulated 5^th^, 50^th^ and 95^th^ percentiles (n=2,000).


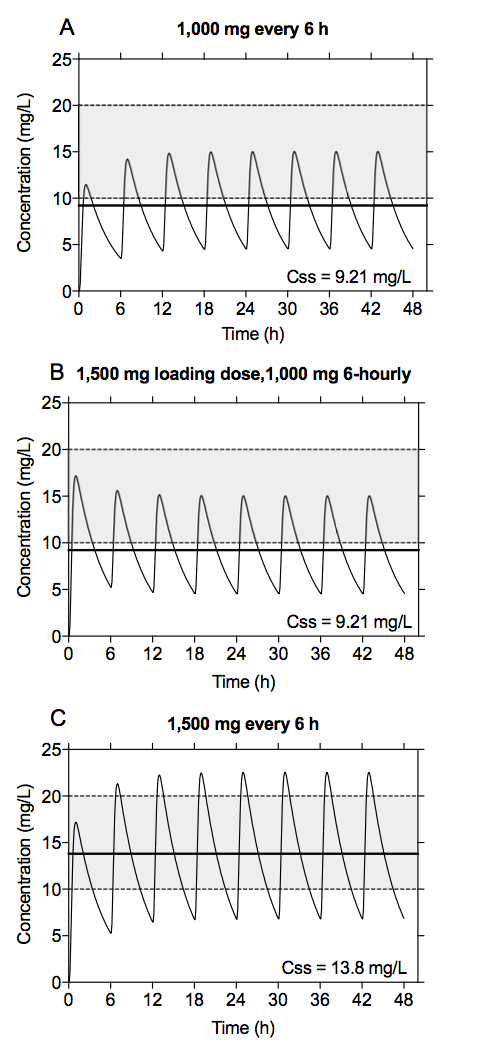


**Supplementary Figure 12.** Simulations from the final population pharmacokinetic model for acetaminophen. Dosing simulations: (A) 1,000 mg every 6 hours; (B) 1,500 mg loading dose followed by 1,000 mg every 6 hours; and (C) 1,500 mg every 6 hours. Dashed lines represent the upper and lower therapeutic plasma concentration range of acetaminophen for fever control (10­-20 mg/L). Solid line represents the steady-state plasma acetaminophen concentration with corresponding numeric value of steady-state achieved (C_ss_).


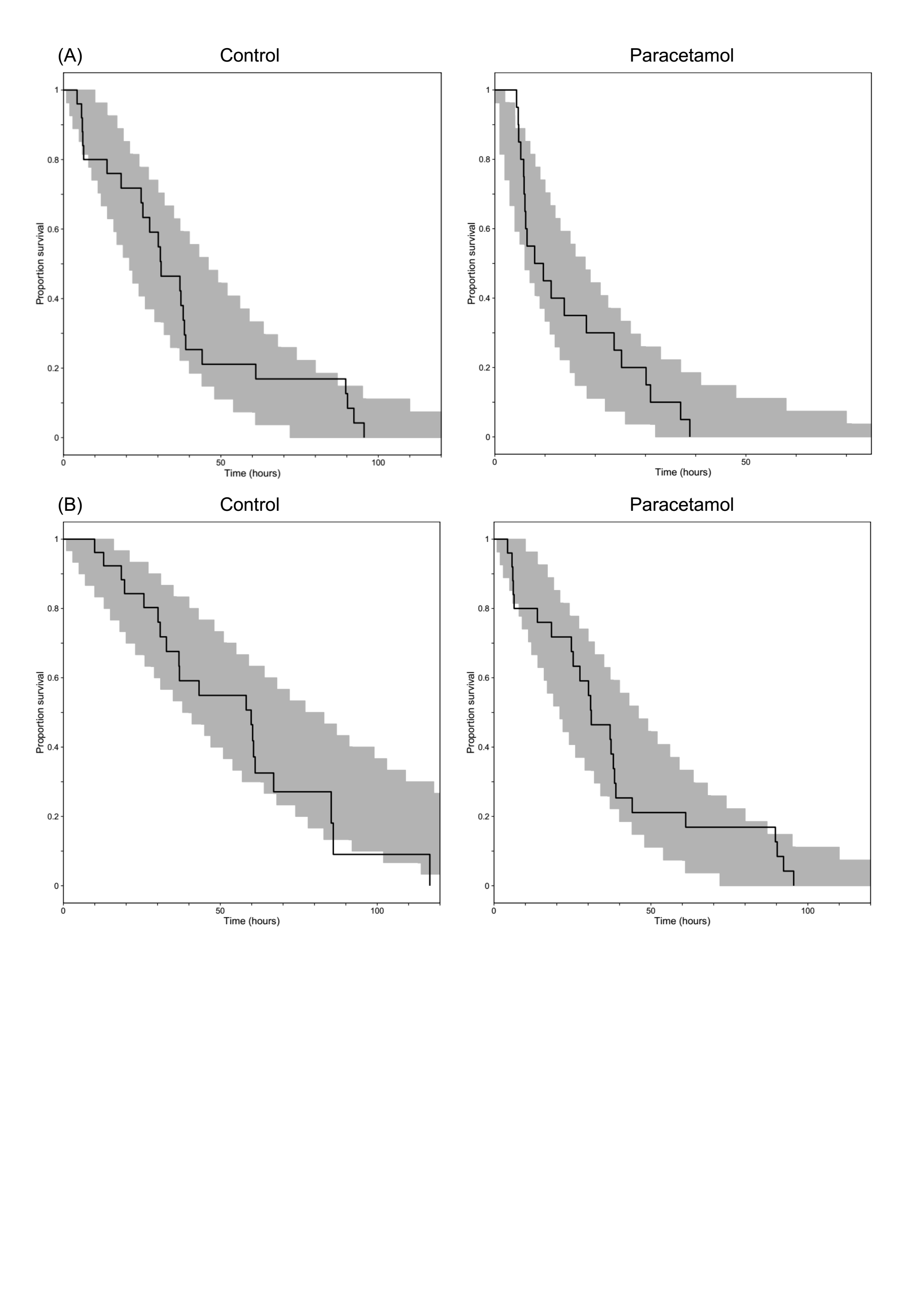


**Supplementary Figure 13.** Kaplan-Meier visual predictive check (n=1,000) of the time-to-event models. (A) Fever clearance time A (FCT-A); and (B) Fever clearance time B (FCT-B) in control and acetaminophen groups. The observed time to achieve the event (FCT-A, and FCT-B) was overlaid with the 95% prediction interval of the simulated time to achieve the event. Abbreviations: FCT-A, fever clearance time A; FCT-B, fever clearance time B.

**REFERENCES**

1. Tun KM, Imwong M, Lwin KM, et al. Spread of artemisinin-resistant *Plasmodium falciparum* in Myanmar: a cross-sectional survey of the K13 molecular marker. Lancet Infect Dis **2015**; 15:415–21.

2. Jonsson EN, Karlsson MO. Xpose--an S-PLUS based population pharmacokinetic/pharmacodynamic model building aid for NONMEM. Comput Methods Programs Biomed **1999**; 58:51–64.

3. Keizer RJ, van Benten M, Beijnen JH, Schellens JH, Huitema AD. Pirana and PCluster: a modeling environment and cluster infrastructure for NONMEM. Comput Methods Programs Biomed **2011**; 101:72–9.

4. Lindbom L, Ribbing J, Jonsson EN. Perl-speaks-NONMEM (PsN)--a Perl module for NONMEM related programming. Comput Methods Programs Biomed **2004**; 75:85–94.

5. Savic RM, Jonker DM, Kerbusch T, Karlsson MO. Implementation of a transit compartment model for describing drug absorption in pharmacokinetic studies. J Pharmacokinet Pharmacodyn **2007**; 34:711–26.

6. Zahnley T, Gittelsohn M. Berkeley Madonna. Modeling and Analysis of Dynamic Systems. 8.3.21 ed. California: University of California at Berkeley, CA, USA, **2000**.

7. Gibb IA, Anderson BJ. Paracetamol (acetaminophen) pharmacodynamics: interpreting the plasma concentration. Arch Dis Child **2008**; 93:241–7.

8. Rumack BH. Aspirin versus acetaminophen: a comparative view. Pediatrics, **1978**; 62: 943-6.

9. World Health Organization. Severe malaria. Trop Med Int Health **2014**; 19 Suppl 1:7–131.

10. Hillmen P, Hall C, Marsh JC, et al. Effect of eculizumab on hemolysis and transfusion requirements in patients with paroxysmal nocturnal hemoglobinuria. N Engl J Med **2004**; 350:552–9.

11. Chotivanich K, Udomsangpetch R, McGready R, et al. Central role of the spleen in malaria parasite clearance. J Infect Dis **2002**; 185:1538–41.
